# The East Siberian Arctic Shelf: towards further assessment of permafrost-related methane fluxes and role of sea ice

**DOI:** 10.1098/rsta.2014.0451

**Published:** 2015-10-13

**Authors:** Natalia Shakhova, Igor Semiletov, Valentin Sergienko, Leopold Lobkovsky, Vladimir Yusupov, Anatoly Salyuk, Alexander Salomatin, Denis Chernykh, Denis Kosmach, Gleb Panteleev, Dmitry Nicolsky, Vladimir Samarkin, Samantha Joye, Alexander Charkin, Oleg Dudarev, Alexander Meluzov, Orjan Gustafsson

**Affiliations:** 1 International Arctic Research Center, University of Alaska Fairbanks, Akasofu Building, Fairbanks, AK 99775-7320, USA; 2 Tomsk Polytechnic University, Institute of Natural Resources, Geology and Mineral Exploration, 30 Prospect Lenina, Tomsk, Russia; 3 Russian Academy of Sciences, Pacific Oceanological Institute, 43 Baltiiskaya Street, Vladivostok 690041, Russia; 4 Russian Academy of Sciences, Institute of Chemistry, 159, 100-Let Vladivostok Prospect, Vladivostok 690022, Russia; 5 Russian Academy of Sciences, P.P. Shirshov Oceanological Institute, 36 Nahimovski Prospect, Moscow 117997, Russia; 6 Russian Academy of Sciences, Institute on Laser and Information Technologies, 2 Pionerskaya Street, Troitsk 142092, Russia; 7 University of Alaska Fairbanks, Geophysical Institute, Snow, Ice and Permafrost, PO Box 757320, Fairbanks, AK 99775-7320, USA; 8 Department of Marine Science, University of Georgia Atlanta, 3475 Lenox Road, NE Suite 300, Atlanta, GA 30326-3228, USA; 9 Department of Applied Environmental Science and Bolin Centre for Climate Research, Stockholm University, Stockholm 10691, Sweden

**Keywords:** methane emissions, subsea permafrost, sea ice, East Siberian Arctic Shelf

## Abstract

Sustained release of methane (CH_4_) to the atmosphere from thawing Arctic permafrost may be a positive and significant feedback to climate warming. Atmospheric venting of CH_4_ from the East Siberian Arctic Shelf (ESAS) was recently reported to be on par with flux from the Arctic tundra; however, the future scale of these releases remains unclear. Here, based on results of our latest observations, we show that CH_4_ emissions from this shelf are likely to be determined by the state of subsea permafrost degradation. We observed CH_4_ emissions from two previously understudied areas of the ESAS: the outer shelf, where subsea permafrost is predicted to be discontinuous or mostly degraded due to long submergence by seawater, and the near shore area, where deep/open taliks presumably form due to combined heating effects of seawater, river run-off, geothermal flux and pre-existing thermokarst. CH_4_ emissions from these areas emerge from largely thawed sediments via strong flare-like ebullition, producing fluxes that are orders of magnitude greater than fluxes observed in background areas underlain by largely frozen sediments. We suggest that progression of subsea permafrost thawing and decrease in ice extent could result in a significant increase in CH_4_ emissions from the ESAS.

## 1. Introduction

The Arctic seabed is believed to contain a significant pool of organic carbon and methane (CH_4_) preserved within and beneath the subsea permafrost, including permafrost-related and continental slope CH_4_ hydrates [1–3]. Sustained CH_4_ release to the atmosphere from thawing Arctic permafrost and dissociating hydrates were suggested to be positive and likely to be significant feedbacks to climate warming [4,5]. Some authors believe that CH_4_ fluxes from subsea permafrost, more than 80% of which occur in the East Siberian Arctic Shelf (ESAS), will depend on rates of CH_4_ production in gradually thawing sediments [6], while subsea permafrost will remain frozen for millennia [7]. Others believe that permafrost failure caused by long-lasting warming by seawater due to sea-level rise and global-change-induced warming, which in the twenty-first century is very pronounced over the ESAS [8], will destabilize massive gas reservoirs, leading to large-scale CH_4_ releases, including release of pre-formed CH_4_ long preserved within and beneath subsea permafrost [9,10].

Our ability to project future CH_4_ emissions from the ESAS largely depends on understanding the relationship between existing CH_4_ fluxes and environmental features that control these fluxes. CH_4_ releasing to the water column could result from modern methanogenesis and/or could originate from seabed deposits (that is, accumulations of pre-formed CH_4_, preserved as free gas and/or hydrates) [11,12]. CH_4_, produced within marine sediment and accumulated in the pore water as dissolved CH_4_, usually does not reach the water column because it is oxidized in the sulfate reduction zone; this does not apply to CH_4_ releasing as bubbles, because biogeochemical filter is only effective on dissolved CH_4_ [13]. In areas of the World Ocean where the organic carbon content of sediments is high or seawater is polluted with human waste, CH_4_ production rates could exceed the capacity of the sulfate reduction zone and CH_4_ could be released to the water column [13].

To attribute CH_4_ fluxes to the current state of subsea permafrost, we aimed to assess the range of modern fluxes, observed over the entire area of the ESAS and compare these rates with the current state of subsea permafrost in different areas of the ESAS. To do so, we needed to evaluate fluxes from earlier underestimated areas such as outer shelf with water depth more than 50 m, where permafrost has presumably degraded the most according to modelling results, because it was submerged by seawater ≈10–15 000 years ago at the beginning of the Holocene [14]. If this assumption about the current state of subsea permafrost were true, CH_4_ flux from these areas would represent the maximum possible CH_4_ flux in the ESAS and indicate the potential for flux increase if ESAS permafrost thawing progresses. Another fraction of the ESAS that lacked our attention previously was the near shore area that was inundated most recently (less than 1000 years ago).

Most recently developed subsea permafrost models, which incorporated sediment salinity, parametrized unfrozen water content and the influence of preceding thermokarst (that is, the hummocky landscape left behind when ice-rich permafrost melts), have shown that taliks (that is, layers or columns of thawed sediments within permafrost), developed over the ESAS at the beginning of the Holocene, might not have frozen after their submergence by seawater [15,16]. Thus, we included one such area in our investigation (Ivashkina Lagoon), where some authors suggested the existence of a deep talik before inundation [17]. Because the water column in the ESAS is very shallow (mean depth approx. 50 m), it provides a very short path for bubble-transported CH_4_ to escape to the atmosphere. However, in deeper waters, a significant fraction of bubbles will dissolve and remain in the water column. Turnover time of dissolved CH_4_ will depend on rates of oxidation by methanotrophic bacteria. As the residence time of seawater on the ESAS shelf could be shorter than the turnover time of dissolved CH_4_, it could be transported laterally to other parts of the Arctic Ocean (AO). Therefore, it is important to elucidate the fate of dissolved CH_4_ in the ESAS.

## 2. Study area and methods

### (a) Study area

**Figure 1.**
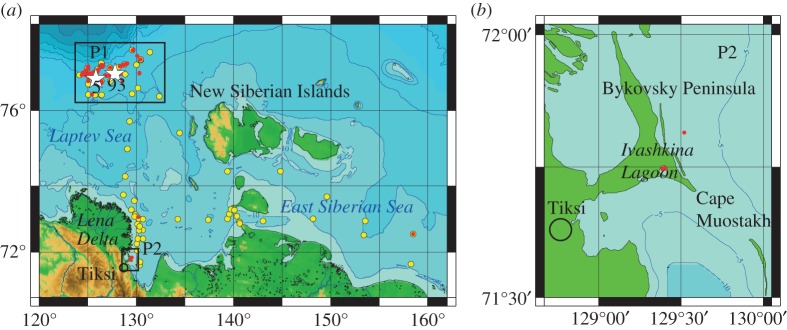
Study area. (*a*) Black rectangles mark the position of polygon 1 (P1, outer shelf) and polygon 2 (P2, near shore area); red circles show the position of discovered seep fields in the study area where hydro-acoustical investigations were performed: white stars mark the position of two seep fields (F5 and F93) where detailed surveys were performed; yellow circles show the position of oceanographic stations in the study area; green shows land; blue shows water; bathymetry lines are shown as black counters; (*b*) the red circle shows position of the Ivashkina Lagoon within P2.

This study covered two areas of the ESAS (figure 1): the ice-free area of the Laptev Sea (between 76.5–77.5° N and 121–132° E, water depth between 50 and 165 m, total area (*S*) of approx. 6400 km^2^, polygon 1, P1), and the near shore area in the southeastern part of the Laptev Sea (between 71–74° N and 129–131° E, water depth less than 20 m, total area approx. 2500 km^2^, polygon 2, P2). The former area was chosen as representative of the outer shelf, where permafrost thawing was suggested to be largely complete based on modelling results [14,16]. The latter area was assumed to be representative of the near shore zone affected by thermokarst, where the possible existence of taliks was suggested due to increased fault-related geothermal flux and/or river heat-induced flux and/or thermokarst progression after submergence [16]. New observational data were collected during three summer campaigns in September–October 2011, 2012 and 2013, and two winter surveys in April 2011 and 2012.

### (b) Sea ice and polynyas

Analysis of datasets collected on sea ice extent (SIE) in the Siberian seas (1932–2005) revealed a significant SIE decrease during the entire period of observations: area-averaged mean monthly ice thickness in perennial ice pack in the basin decreased by 1.1 m (from 2.7–3.1 m to 1.4–1.9 m) [18]. Because ice thickness in the AO depends on air temperature and ice dynamics, it was suggested that the warming might most affect the thickness of the fast ice [19]. A specific feature of the ESAS is that during the winter, about 10% of the shelf area is composed of open water (and young ice off the land-fast ice) or polynyas, which are parts of the Great Siberian Polynya [20]. Followed by land-fast ice formation, polynyas develop in November when wind breaks apart the fast ice and the drifting sea ice propagates hundreds of kilometres offshore [21].

### (c) Seep detection

Bubbles in the water column usually come from seeps on the sea floor and could be detected using backscattered images of bubbles because there is a pronounced acoustic impedance difference between water and bubbles [22]. Bubbles usually escape via venting holes on the sea floor, which may or may not be surrounded by bacterial mats. In this research, we define seep as an area on the sea floor where CH_4_ releases from a single bubble-sized vent (electronic supplementary material, figure S1); such bubbles create a single-track image on acoustic echograms [10]. In places, bubbles release as a vigorous flow that often reach the sea surface; on echograms, such bubble plumes create specific flare-like images. Seeps could exhibit sparse occurrence on the sea floor or could be observed as relatively large and dense areas, where numerous single-bubble seeps occur (seep field); when a seep field includes flares, we define such an area a flare seep field. To detect, map, monitor and analyse seep fields in summer 2011, a multi-channel hydro-acoustic complex was designed using one hull-mounted ELAC single-beam echo sounder and two Sargan echo sounders on the RV Academician Lavrent'ev with triple-frequency 12, 20 and 135 kHz transducers covering 12°, 10° and 4° beam width, respectively. In summer 2012, sonar data were gathered using a SIMRAD EK15 SW 1.0.0 echo sounder (www.simrad.com) with 200 kHz operational frequency, 80–1240 μs pulse duration, 26° beam width and built-in calibration system. Seep and flare-imaging data were recorded at an average survey speed of 4–6 knots. The backscattered signal was digitally recorded, visualized and processed using an original software package provided by SIMRAD EK 15 (EchoView and Sonar5). The backscattering strength of the bubbles was measured at a frequency of 200 kHz with a pulse length of 1 ms repeated every 100 ms. To measure the bubble screen backscattering strength, the acoustic sensors were calibrated using a target provided by the manufacturer.

### (d) Visual and hydro-acoustical observation of bubbles

Bubbles were observed during calm weather conditions using a submerged cabled remotely operated vehicle (ROV) equipped with a high-speed high-resolution video camera. During the ROV dives, several individual seep outlets were observed, in which bubbles were released distinctly one by one. Each outlet was observed in detail for 20 min to 1 h. To resist currents and stabilize video recording and camera operation, the ROV was attached to the Rosette frame. The Rosette equipped with Niskin bottles was deployed to the sea floor and left in place for about 1 h, which allowed bubble sampling and bubble image recording, followed by statistical treatment of records. Bubble sizes were calculated relative to the sizes of particular parts of the Niskin bottles, which were marked with measuring tape, and treated statistically to obtain observed frequencies (electronic supplementary material, figure S2).

### (e) Quantification of methane fluxes from the seafloor

To quantify CH_4_ fluxes conveyed by bubbles releasing from the seafloor, we implemented an approach in which we aimed to combine the advantage of accuracy that could be achieved by evaluating CH_4_ flux using direct seep observations, with the advantage of wide area coverage, which was achieved by collecting bubble-imagery sonar data. To interpret sonar data, we performed an *in situ* calibration using a method described in detail in [23]. Calibration aimed to establish a relationship between the backscattering strength of CH_4_ bubbles and gas flux rate. Results achieved by use of *in situ* calibration were validated by comparing these results with estimates obtained based on direct *in situ* observations of CH_4_ bubble flow.

Implementation of this approach included a few steps. First, we detected areas of seep fields within the studied area using single-beam sonar. Then, we classified these seep fields by area size and strength of the backscattering signal recorded by sonar. After that, we focused on detailed surveys of randomly chosen seep fields to evaluate the number of individual seeps in each seep field. Several such seeps, in which bubbles were distinctly emitted from the seafloor one by one, were chosen for continuing observation to obtain bubble sizes and rates of bubble release per unit of time. For each seep, mean CH_4_ flux was calculated by multiplying mean bubble volume (assuming 100% CH_4_ content) by mean bubble release rate observed during the time of observation. Finally, we assessed bubble-induced CH_4_ flux using the results of *in situ* sonar calibration of sonar data (electronic supplementary material, figure S3).

For calibration, we used nitrogen (N_2_) as a calibration gas (40 l volume, 460 atm). The tank was installed on the foredeck of our research vessel. PVC pipe, 12 mm in diameter, 6 mm wall thickness, 70 m long, was attached to Kevlar wire with a heavy metal load (about 30 kg) on the end and was deployed to a depth of 40 m where the water depth was 45 m. The multi-beam and single-beam sonars were located near each other so that their beam coverage overlapped, but the centre points of the beam diagrams focused on the bubble stream produced using a copper nozzle 4 mm in diameter attached to the end of the PVC pipe. Gas flux was tuned using a standard flow meter. One port of a flow meter was connected to a PVC pipe, the second port was connected to the gas tank through the gas reduction system, which consisted of one high-pressure sensor to measure the pressure remaining in the tank and one low-pressure sensor to measure the emitted pressure (5.5 atm). Gas flow changed from 0.5 to 150 l min^−1^. Measurements started after the gas flow stabilized and lasted about 10 min for each session. We conducted eight sessions in the chosen location. No natural seepages occurred in this place and the sea floor was almost flat, excluding (or at least reducing) the possibility of scattering from the bottom. Wind during the sessions was 1–3 m s^−1^ and there were almost no waves. Conductivity–temperature–depth (CTD) data were obtained for the site using an SBE19plus CTD probe (USA). The vessel was anchored during the calibration session. *In situ* calibration of an operational system allowed the measured echo level of a known acoustically insonified bubble volume to be directly related to the bubble flux rate (electronic supplementary material, tables S1 and S2). The final step aimed to estimate the total number of seeps in the studied area by estimating seep density, which allowed interpolation of CH_4_ fluxes within the studied area.

### (f) Calculation of seep field density

Seep density is the spatial distribution of the seeps per square unit of the seep field area (electronic supplementary material, figure S4). To establish correlation between the backscatter value and the numbers of acoustically detected seeps/flares per m^2^, we used a method described in detail in [24]. Because we detected seeps using the single-beam echo sounder, to establish a correlation we considered the area that was actually insonified by the system. The size of the examined area was estimated as the total length of the vessel's path over the sections of the examined area multiplied by the width of the zone sounded by the echo sounder (the diameter of the circle bounded by the beam width of the echo sounder transducer). The total number of seep fields was determined by multiplying seep field density by the study area (more details could be found in the electronic supplementary material).

### (g) Spatial distribution of organic carbon content (*C*_org_)

*C*_org_ data were obtained from surface sediments sampled by Van Veen grab at more than 700 sites in the ESAS over the 2003–2009 period. The study areas visited in different years are presented in [25]. Concentrations of *C*_org_ in surface sediment were measured at the University of Alaska Fairbanks using a Finnigan isotope ratio mass spectrometer as described in detail in [26].

### (h) Methane oxidation rates

CH_4_ oxidation rates were measured in the water column, using a C_3_H_4_ radiotracer following the procedure described in [27]. The CH_4_ oxidation rate constant was calculated following [27] and multiplied by *in situ* CH_4_ concentration to determine the CH_4_ oxidation rate (more details could be found in the electronic supplementary material).

#### (i) Subsea permafrost modelling

The thermodynamic model of soil freezing/thawing, which already partially incorporated thermokarst and land–ocean interaction theory [14], was forced by seawater temperature dynamics in the ESAS and computed by GCMs. The permafrost was simulated using the 100-year mean benthic temperature; the local seawater warming effect from the Lena River discharge was incorporated using original data as described in [10]. To compute temperature dynamics at sites within tectonics fault zones, we used a 2D realization of the thermodynamic model, which allows simulation of open talik formation and evolution. The systematic variability associated with horsts and grabens was characterized by a 2D Fourier series. To compute temperature dynamics in time, a finite-element scheme, backward Euler in time, and based on the enthalpy technique for the solution of Stefan-type problems, was used.

#### (ii) Modelling lateral transport of dissolved methane on the East Siberian Arctic Shelf

The climatological circulation in the AO during 1997–2006 was reconstructed by assimilating oceanic and surface heat data into the Semi-Implicit Ocean Model [28,29] with 26×26 km resolution using the 4D variational approach [30]. The reconstructed annual velocities at 12.5 m water depth were used to calculate the trajectories of several groups of Lagrangian particles (molecules of dissolved CH_4_) using the standard Runge–Kutta fourth-order-accuracy algorithm [31]. Transport of the particles was integrated for periods of 2 and 3 years, which is in accordance with turnover time of the dissolved CH_4_ pool in the ESAS (more details could be found in the electronic supplementary material).

## 3. Results

### (a) Results of seep detection over the study area

**Figure 2.**
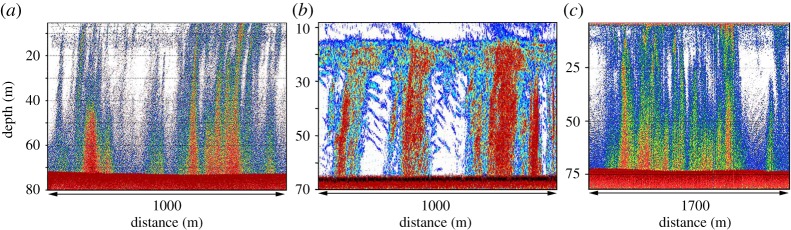
Hydro-acoustical images of detected seep fields including flares observed in P1 (September–October 2011). (*a*) Medium seep fields including flares (MF); (*b*) Large seep fields associated with large flares (LF). (*c*) Small seep field associated with small flares (SF).

We observed a high concentration of bubble seeps in P1, where we detected large seep fields of up to 700 individual seeps, including flares (figure 2). Not all seep fields were subjected to detailed observation by the deployed ROV, but we observed no seeps surrounded by bacterial mats. That might indicate that bubble flux is the predominant source of CH_4_ in the sites and the supply of dissolved CH_4_ is insufficient to support bacterial communities. We observed 112 flare seep fields located in the 50–90 m depth range. Statistical testing of the datasets, which included calculated areas of seep fields, allowed us to define three classes of seep fields: small seep field (SF), medium seep field (MF) and large seep field (LF) seep fields with mean areas of 85.2 m^2^, 2.2×10^4^ m^2^ and 4.2×10^4^ m^2^, respectively (electronic supplementary material, table S1).

### (b) Methane flux by direct bubble observation

Based on video recording, we estimated the amount of CH_4_ released by bubbles escaping from the seafloor at different seep sites. To assess bubble sizes, we analysed more than 1000 records of bubbles, the radii of which varied from 1 to 10 mm with the greater fraction (more than 70%) being in the range of 3–6 mm with mean radius of 4 mm (electronic supplementary material, figure S2). During the observed period, bubble release from single vents varied from 1.5 to 5.7 bubbles s^−1^; for approximately 50% of the time fluxes remained steady, but then they ceased or increased; in a few seeps, fluxes stopped for approximately 30% of the observed time but then started up again. For our estimates, we assumed steady flux maintained approximately 50% of the time by 3.6 bubbles s^−1^ of 4 mm in radius, giving a mean flux of 0.044 mmol-CH_4_ s^−1^, corresponding to 3.4 mol-CH_4_ d^−1^ or 54.4 g d^−1^ from one vent. This implies that areal flux would vary depending on the number of vents within the seepage area, which might change from tens to hundreds in one seep field.

### (c) Methane fluxes by absolute calibration

To approach area-weighted fluxes, we estimated the density of the seep fields in the study area. First, we established a relationship between seep field occurrence along the ship's path and the area covered by a sonar beam in a single survey. To minimize uncertainty while establishing a correlation between backscatter value and seep occurrence, we performed in-depth investigation of a few seep fields in order to achieve the densest area coverage possible in particular field conditions (electronic supplementary material, figure S4). For example, in the 5.76 km^2^ F5 seep field, 2.3 km^2^ was actually insonified, which represents 40% of the total seep field area; in the 9.8 km^2^ F93 seep field, we achieved 13% coverage of the entire seep field area. The size of the examined area was estimated by multiplying the total length of the vessel's path over the sections of the examined area by the width of the zone sounded by sonar. Density was then estimated by dividing the size of the examined area by the number of observed seep fields of a certain size. We calculated mean CH_4_ fluxes from SF, MF and LF seep fields to be 30.8, 88 and 176 g CH_4_ m^−2^ d^−1^, respectively. Using estimated density, integrated minimum CH_4_ flux to the water column in the P1 was estimated to be 1.94×10^10^ g CH_4_ d^−1^ (electronic supplementary material, table S2).

These results suggest that estimates based on calibration curves are more conservative than those performed based on direct observations of bubbles, because the radii of the observed bubbles were larger than were those used in calibration. Another reason could be that when bubbles are released as large streams, they might interact with each other creating acoustical coupling; this could potentially decrease the sonar return signal (backscatter) [32]. A number of uncertainties exist in the quantitative assessment of bubble-transported CH_4_ fluxes, because the gas exchange between bubbles and water depends on bubble size, shape, bubble rise velocity as well as on varying properties of seawater [33]. One such uncertainty springs from the fact that the ability of single-beam sonar to capture seeps outside the area covered by the beam is limited. To minimize this uncertainty, coverage of 13–40% was achieved during in-depth studies of chosen seep fields (electronic supplementary material, figure S4). Another uncertainty is related to the sporadic nature of seeps and flares, which causes high temporal variability of CH_4_ fluxes [23,24]. When acoustic targets are highly concentrated (like bubbles in plumes), a shadowing effect might occur [22]. To eliminate or minimize the shadowing effect, some authors [32,33] have suggested using frequencies higher than 50 kHz and calibrating the sonar system using gas emitted at known flux rates that was implemented in our study.

**Figure 3.**
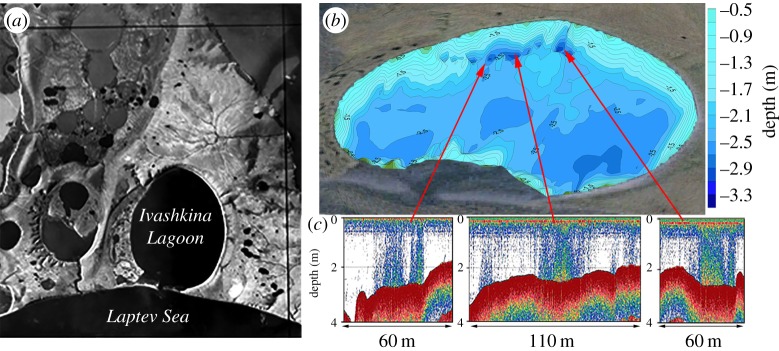
Results of CH_4_ flux observations in the Ivashkina Lagoon (October 2013). (*a*) The former thermokarst lake (mean depth less than 2 m) is transforming into a sea lagoon; (*b*) bubble releases in the lagoon occur from the steep narrow depressions observed in the north part of the lagoon; (*c*) CH_4_ releases occur from shallow depths and reach the atmosphere. Estimated rates of these releases are from 5.5 to 24 g m^−2^ d^−1^.

In October 2013, we performed observations in the southernmost part of P2 (figure 1), in Ivashkina Lagoon, which has been progressively inundated during the last approximately 100–200 years, replacing a former thermokarst lake (figure 3). The importance of this investigation is in that according to an existing assumption (which is one of the most important in permafrost modelling), submerged thermokarst lakes, which widely developed over the ESAS in the beginning of the Holocene, become frozen after submergence; therefore, no gas release should have occurred from that lagoon [14,17]. Despite that assumption, we observed vigorous bubble release from narrow and steep depressions aligned parallel to the lagoon's northern edge. Backscattering cross-sections of the bubbles emitted from 17 seeps observed in the Ivashkina Lagoon were recorded for 36 h using portable single-beam sonar, which was calibrated *in situ* during the same campaign. In the Ivashkina Lagoon, CH_4_ fluxes observed in October 2013 ranged from 5 to 24 g m^−2^ d^−1^ (averaged over the total area of 3000 m^2^). Our observations demonstrate that understanding of the process of permafrost degradation and associated permeability of permafrost for gases after submergence needs to be improved.

### (d) Fraction of methane reaching the sea surface

To assess what fraction of CH_4_ bubbles reaches the sea surface, we performed experimental work from the fast ice in April 2013 in the southern part of P2. We drilled a hole in the sea ice and created an artificial seep at approximately 6 m water depth as described above. A gas tank was installed on the fast ice. By tuning the valve (changing the pressure) installed on the gas tank head, bubble flow controlled by a flowmeter was changed from 0.2 to 2.0 l min^−1^ by creating a flow of approximately 5 mm diameter bubbles. We captured these bubbles escaping from the water surface using a chamber installed over the hole in the sea ice. After 1 h of exposure, we examined the composition of gas collected in the chamber and measured actual CH_4_ flux from the water surface. Our data show that at a shallow water depth, approximately 67–72% of CH_4_ remains in the bubbles when the bubbles reach the sea surface (electronic supplementary material, table S3). This assessment is only applicable to shallow water depths; to assess the fraction of CH_4_ that reaches the surface from deeper water, there is a need to perform additional investigations of bubble plume dynamics in the water column.

### (e) Fate of methane released to the water column

**Figure 4.**
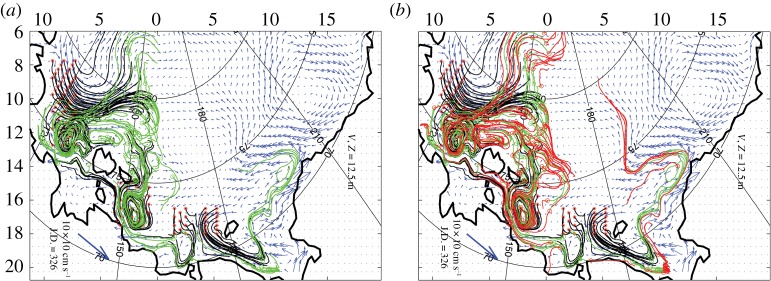
Reconstructed velocity fields and trajectories of the passive tracer particles (dissolved CH_4_ molecules) launched in the ESAS. (*a*) Results of a 2 year run (700 days CH_4_ pool turnover time). (*b*) Results of a 3 year run (1000 days CH_4_ pool turnover time). Current velocities are shown as blue arrows; CH_4_ trajectories in the first year are shown as red arrows, in the second year as green arrows, and in the third year as black arrows.

How much of the CH_4_ carried by bubbles will reach the sea surface and be released to the atmosphere largely depends on the CH_4_ flux rate, water depth and *in situ* release conditions that control transfer processes [32,33]. Most of the CH_4_ dissolves in the water column, building up an aqueous CH_4_ inventory. The fate of dissolved CH_4_ largely depends on the interaction between a few factors: the turnover time of dissolved CH_4_ in the water column, the stability of the water column against vertical mixing and the rates of turbulent diffusion and lateral advection. Dissolved CH_4_ in the outer ESAS requires 300–1000 days to be oxidized in the water column because CH_4_ oxidation rates are very low (mean±1 s.d.: 0.0988±0.1343 nM d^−1^, *p*=0.95, *n*=328). During this time, some of the aqueous CH_4_ inventory is likely to be released to the atmosphere during storms [10]. The remaining dissolved CH_4_, captured beneath the sea ice in winter, can spread further from the ESAS via currents (figure 4), and some can escape to the atmosphere through leads and breaks in the ice [34].

## 4. Discussion and conclusion

### (a) Role of the sea ice

Sea ice serves as a natural physical barrier that restricts CH_4_ emissions from the ESAS during the ice-covered period. Because the temperature in the Arctic has increased at twice the rate as in the rest of the globe, and the region is expected to increase an additional 8°C (14°F) in the twenty-first century [3], longer periods of open water and shorter ice-covered periods [35,36] are occurring. Increasing periods of open water implies an increasing number of storm events, when wind speed increases to 15 m s^−1^ or more and the boundary between sea surface and air increases many times due to deep water mixing. Such events have the potential to rapidly ventilate bubble-transported and dissolved CH_4_ from the water column, producing high emission rates to the atmosphere. Because more than 75% of the total ESAS area is less than 50 m in depth, the water column provides bubbles with a very short conduit to the atmosphere. Storms enable more CH_4_ release because they destroy shallow water stratification and increase the boundary between sea surface and air, thus increasing gas exchange across phase boundaries. As a result, bubble-mediated, storm-induced CH_4_ ‘pulses’ force a greater fraction of CH_4_ to bypass aqueous microbial filters and reach the atmosphere [10].

In addition, about 10% of the ESAS remains open water in winter due to formation of flaw polynyas. It was shown that flaw polynyas provide pathways for CH_4_ escape to the atmosphere during the arctic winter [37]. Areas of flaw polynyas in the ESAS increased dramatically (by up to five times) during the last decades, and now exceed the total area of the Siberian wetlands (electronic supplementary material, figure S5). This implies that the ESAS remains an active source of CH_4_ to the atmosphere year-round. Increasing storminess [38–40] and rapid sea-ice retreat [36] causing increased CH_4_ fluxes from the ESAS are possible new climate-change-driven processes. Continuing warming of the AO will strengthen these processes, and the role of the ESAS as a year-round contributor to global CH_4_ emissions will grow over time.

### (b) Implications for future emissions

These new data together with those obtained previously show very high variability of CH_4_ fluxes in the ESAS. On the one hand, this points to a lack of established methods that can be used for quantitative assessment of fluxes, both diffusive and bubble-transported. Indeed, because the major driving parameter in calculations of diffusive fluxes is wind speed, adjustment of climatic winds to actual winds changed the estimated CH_4_ fluxes by two orders of magnitude [41,42]. Quantitative derivation of bubble fluxes remains difficult because seep fields could include flares and a number of factors affect the seabed, water column and sea–air fluxes [24,32,33]. In the ESAS, the water column is very shallow and provides a very short path for bubble-transported CH_4_ to the atmosphere. Because the ESAS is the largest shelf in the World Ocean, the development of methods applicable for estimating bubbling flux from large areas over a relatively short time (the period of open water in the Arctic) is very important. Among the methods used to date, the hydro-acoustical method described in [23] and modified in this study seems to be most suitable for flux estimates over large areas rather than for localized *in situ* observations, which could be more area-specific. *In situ* calibration of an operational system allows the measured echo level of a known acoustically insonified bubble volume to be directly related to the bubble flux rate, reducing the number of system parameters that must be known.

**Figure 5.**
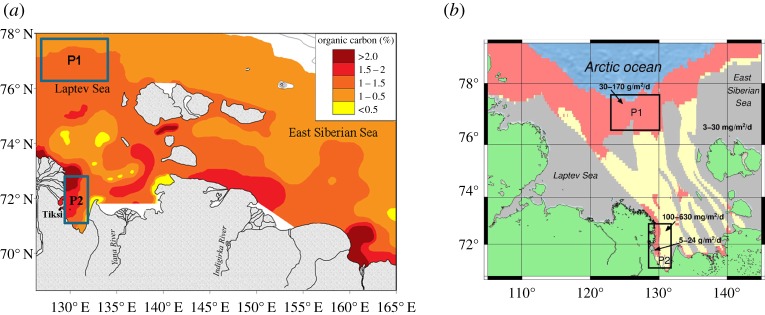
Distribution of total *C*_org_ in the surface sediments versus current state of subsea permafrost and methane (CH_4_) fluxes from the sea floor/sea surface in the ESAS. As seen from panel (*a*), the percentage of *C*_org_ in the surface sediments varies by a factor of 4 (from less than 0.5% to more than 2%) over the ESAS; *C*_org_ content distribution is based on analysis of samples from more than 700 sites visited in the ESAS during 2003–2009. Polygon 1 (P1) is representative of ESAS areas where the *C*_org_ percentage varies from low to moderate levels (less than 0.5–1.5%); polygon 2 (P2) is representative of ESAS areas where the highest *C*_org_ percentage (more than 1.5%) is observed. P1 and P2 are marked with black rectangles. (*b*) Rates of CH_4_ fluxes observed in the ESAS versus results of permafrost modelling. Areas marked in coral represent areas where subsea permafrost is predicted to be exhibiting the most advanced stages of degradation due to duration of inundation; CH_4_ fluxes to the bottom water vary from 30 to 170 g m^2^ d^−1^. Areas marked in yellow represent areas of modelled taliks developing due to geological factors (faults) and warming effect of river discharge; estimated fluxes to the bottom water in these areas vary from 5 to 24 g m^2^ d^−1^; fluxes to the atmosphere in one such area was estimated from 100 to 630 mg m^2^ d^−1^ [10]. Areas marked in blue represent the areas where subsea permafrost presumably remains the least disintegrated; CH_4_ fluxes from these areas vary from 3 mg m^2^ d^−1^ (in background areas) to 30 mg m^2^ d^−1^ (in the hot spots) [9]. Green colour shows the land; orange lines mark the coastline.

On the other hand, these data support the hypothesis that variability of CH_4_ fluxes is determined by the current state of subsea permafrost, which is undergoing destabilization caused by the long-lasting warming effect of inundation by seawater that started at the beginning of the Holocene. Indeed, in the ESAS, organic carbon (*C*_org_) contents of sediments vary by only a factor of approximately 4, while CH_4_ fluxes vary by orders of magnitude (figure 5). Such flux variability could be determined by many factors, including the deep geological structures; however, test results showed that when ice saturation is more than 80%, CH_4_ gas can be completely sealed within the permafrost [43]. This means that when subsea permafrost is ice-bonded and continuous, it is virtually impermeable for mass transfer from geological sources beneath the permafrost [44,45]. Therefore, the state of subsea permafrost is becoming a key factor controlling CH_4_ fluxes from the seabed to the water column in the ESAS.

The range of modern CH_4_ emissions from the seafloor in the ESAS serves as a baseline for monitoring future dynamics in CH_4_ fluxes from the ESAS. We suggest that within the entire range of observed fluxes, the lowest fluxes are associated with an initial degree of subsea permafrost thawing observed in the shallow shelf outside the areas affected by faults, rivers and pre-existing thermokarst. These fluxes are fuelled by modern methanogenesis occurring within sediment accumulations of the Holocene age, which have never been frozen, and/or within partially thawed older sediments beneath them. The highest rates observed over the outer shelf area are likely to represent the maximum emissions, which combine recently produced CH_4_ and long-accumulated pre-formed CH_4_ escaping from seabed deposits through gas migration pathways that are growing in capacity. Shallow hot spots, currently releasing CH_4_ at high rates, are representative of local subsea permafrost disintegration that takes place in areas subjected to development of deep/open taliks due to increased fault-related geothermal flux and/or river heat-induced flux and/or thermokarst progression after submergence.

The observed range in CH_4_ emissions associated with different degrees of subsea permafrost disintegration implies substantial and potent emission enhancement in the ESAS as the process of subsea permafrost thawing progresses with time. While it is still unclear how quickly CH_4_ flux rates will change, the current process of Arctic warming and associated sea ice loss [35,36] will accelerate this process. The potential for the release of substantial amounts of CH_4_ from the ESAS region has important implications not only for atmospheric CH_4_ concentrations but also, given CH_4_'s potency as a greenhouse gas, for the global climate. Because the ESAS contains the largest and arguably most vulnerable stores of subsea CH_4_ [2,10,46,47], inclusion of the ESAS source in global climate models should be considered a high priority.

## Supplementary Material

Supplementary Material includes Supplementary methods, Tables (S1-S3), Supplementary Figures (S1-S6) and Supplementary References (S1-S11)
