# Supplementary material for: The East Siberian Arctic Shelf: towards further assessment of permafrost-related methane fluxes and role of sea ice
Source: Philos Trans A Math Phys Eng Sci. 2015 Oct 13;373(2052):20140451. doi: 10.1098/rsta.2014.0451 (PMC4607703; doi:10.1098/rsta.2014.0451)
Supplement: Supplementary Material includes Supplementary methods, Tables (S1-S3), Supplementary Figures (S1-S6) and Supplementary References (S1-S11) [file rsta20140451supp1.docx]

Supplementary Material

Contents

Supplementary Text

This supplement contains detailed information about the data processing and analysis; a description of hydro-acoustical methods used to detect bubbles releasing from the sea floor; and an estimate of ebullition flux in the study area based on direct observation of in-situ bubble flow and using in*-*situ sonar calibration.

Tables S1-S3

Table S1. Summary of parameters used for CH_4_ flux calculation.

Table S2. Summary of flare seep field class parameters observed in the outer ESAS shelf.

Table S3. Gas composition of bubbles reaching the sea surface in P2.

Supplementary Figures S1-S6

Figure S1. Results of in-situ bubble video-recoding.

Figure S2. Results of bubble analysis.

Figure S3. In-situ sonar calibration data.

Figure S4. Schematic of ship tracks for hydro-acoustical surveys performed in seep fields.

Figure S5. Increase in the areas of flaw polynyas composing the Great Siberian Polynya observed over two decades.

Figure S6. Probability-probability plots.

Supplementary References S1-S14

**Supplementary Text**

**Data processing and analysis**

Evaluation of bubbling CH_4_ flux

The details of the method we used to assess CH_4_ fluxes conveyed by ebullition are described in [S1]. The approach was as follows: 1) single-beam echo-sounder data were used to detect seep fields; 2) echo-sounder data combined with backscatter data were used to extrapolate the total number of seeps in the studied area; 3) bubble fluxes were assessed based on in-situ sonar calibrations; and 4) bubbles were directly observed and recorded using the cabled, submerged remotely operated vehicle (ROV) equipped with a high-speed high-resolution video camera to obtain bubble size and distribution.

Interpretation of the echo-sounding data based on in-situ calibration

We used an echo-integration approach based on determining the bubble screen backscattering strength. An echo level from bubbles may be converted into estimates of the acoustic scattering cross section per unit volume (*m_v_*) of the bubbles by means of the sonar equation in units of dB [S1]:

*M_v_* = *RL – SL + TL -10log(V)* (1)

where *M_v_* =10log(*m_v_*) is the volume backscattering strength, *m_v_* is the integral over the scattering cross section of bubbles inside the unit volume, *RL* is the received level in dB rel µP, *SL* is the source level of the transmitter in dB rel µP at 1 m, and *V* is effective sampled volume, which can be calculated as follows:

ΔV≈
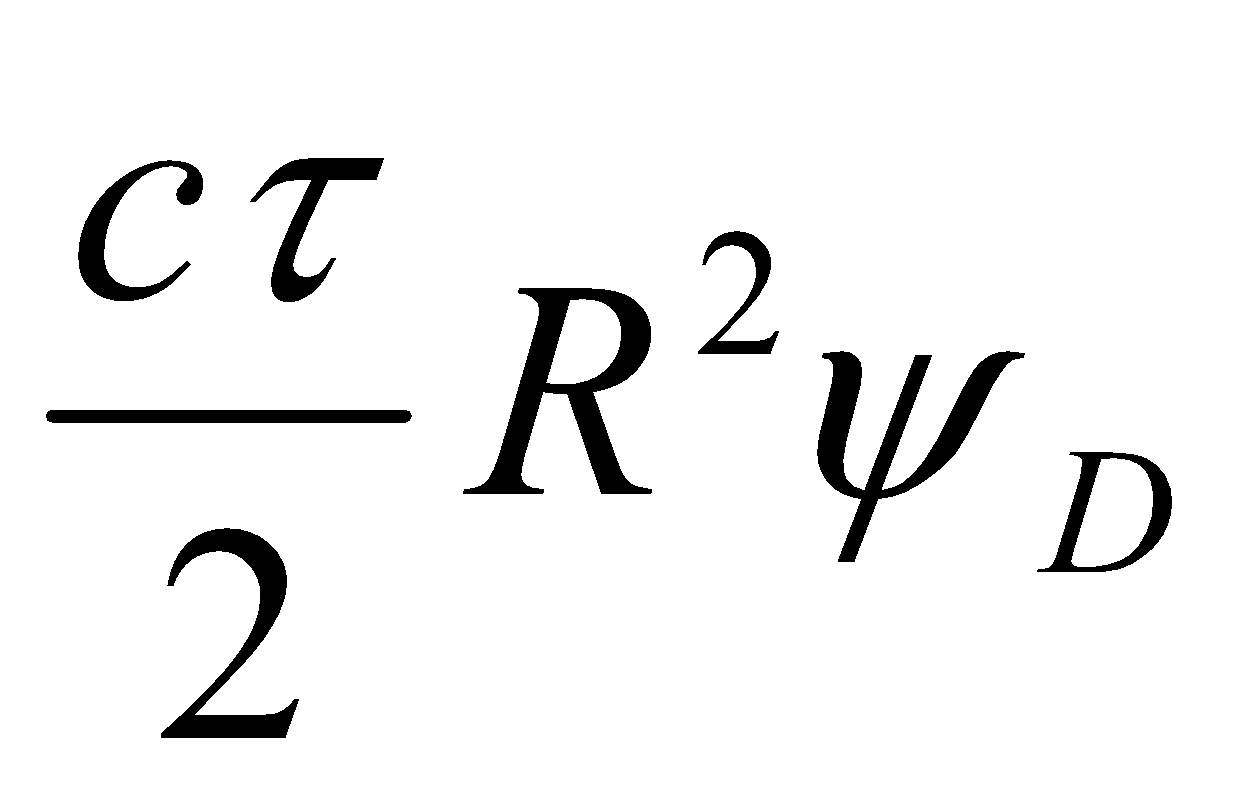
, R>>
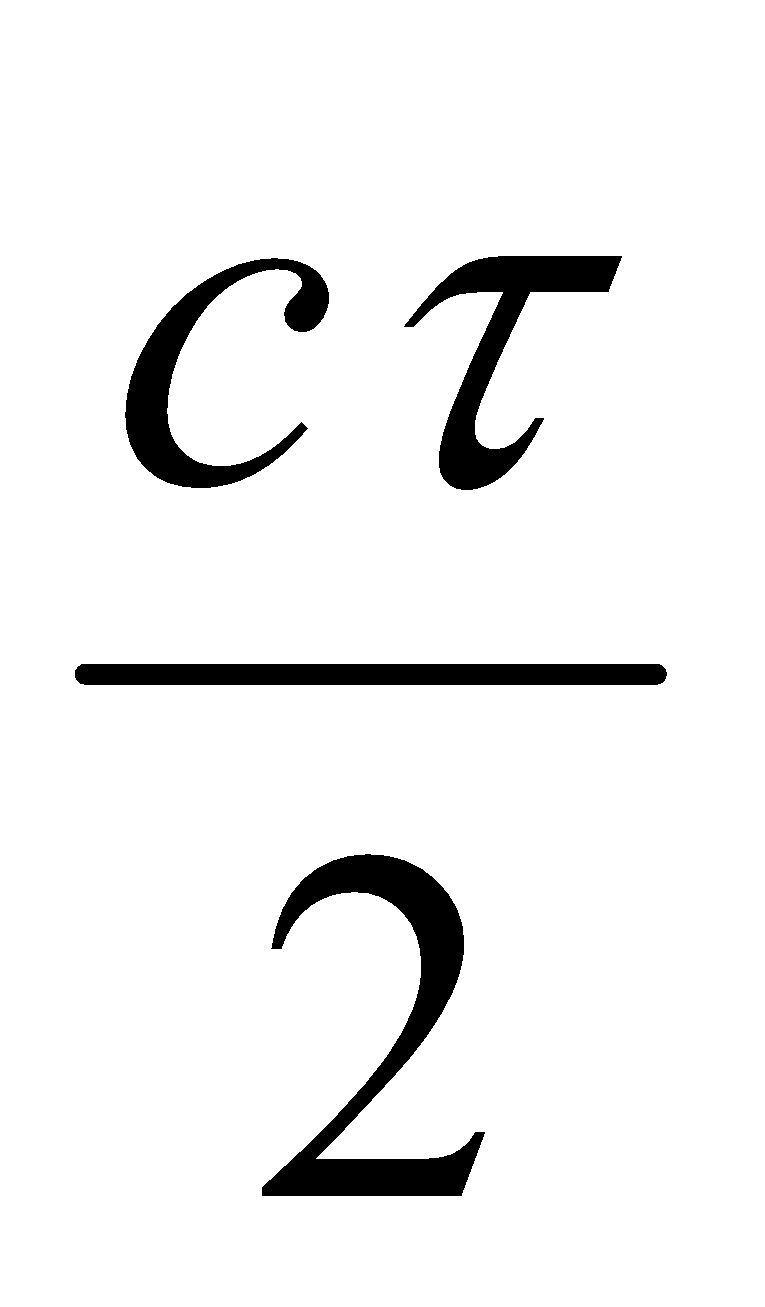
 (2)

where *c* is sound speed, *τ* is pulse duration, R is distance from transducer to scattering volume, and
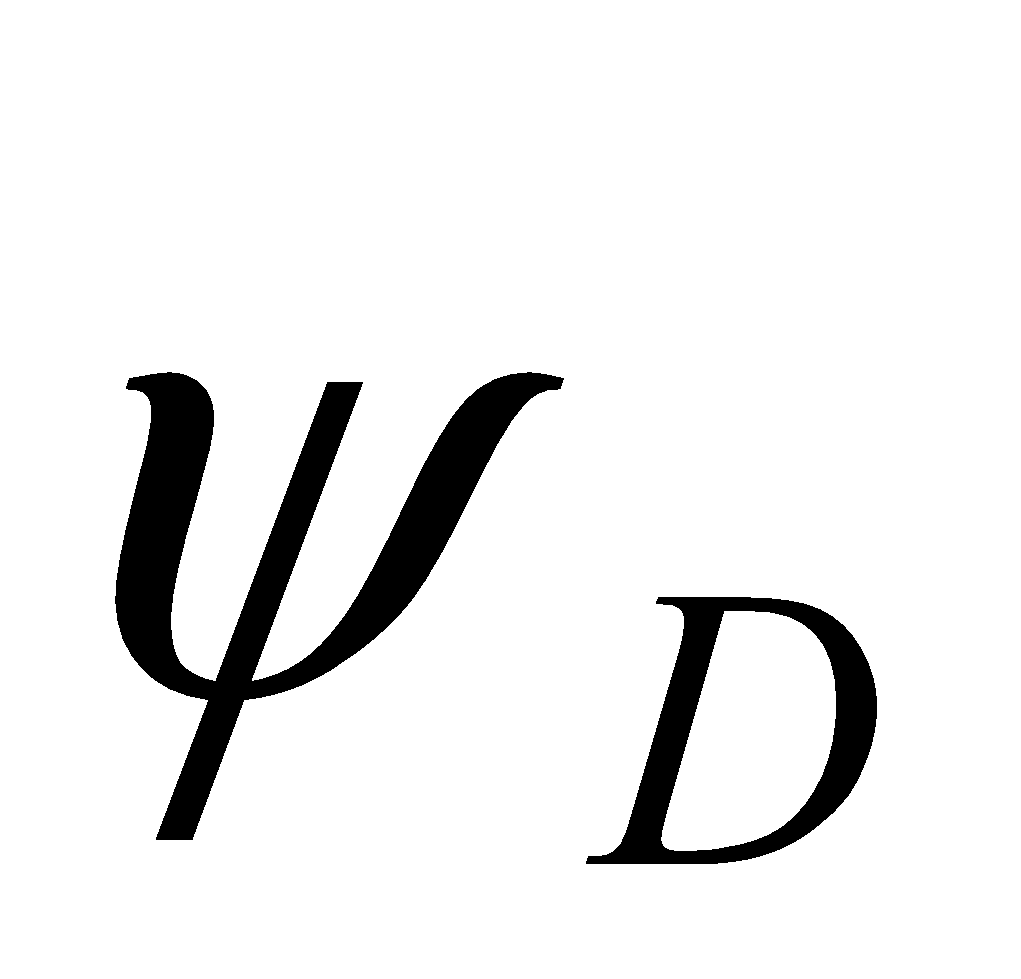
is integrated beam pattern, which is:


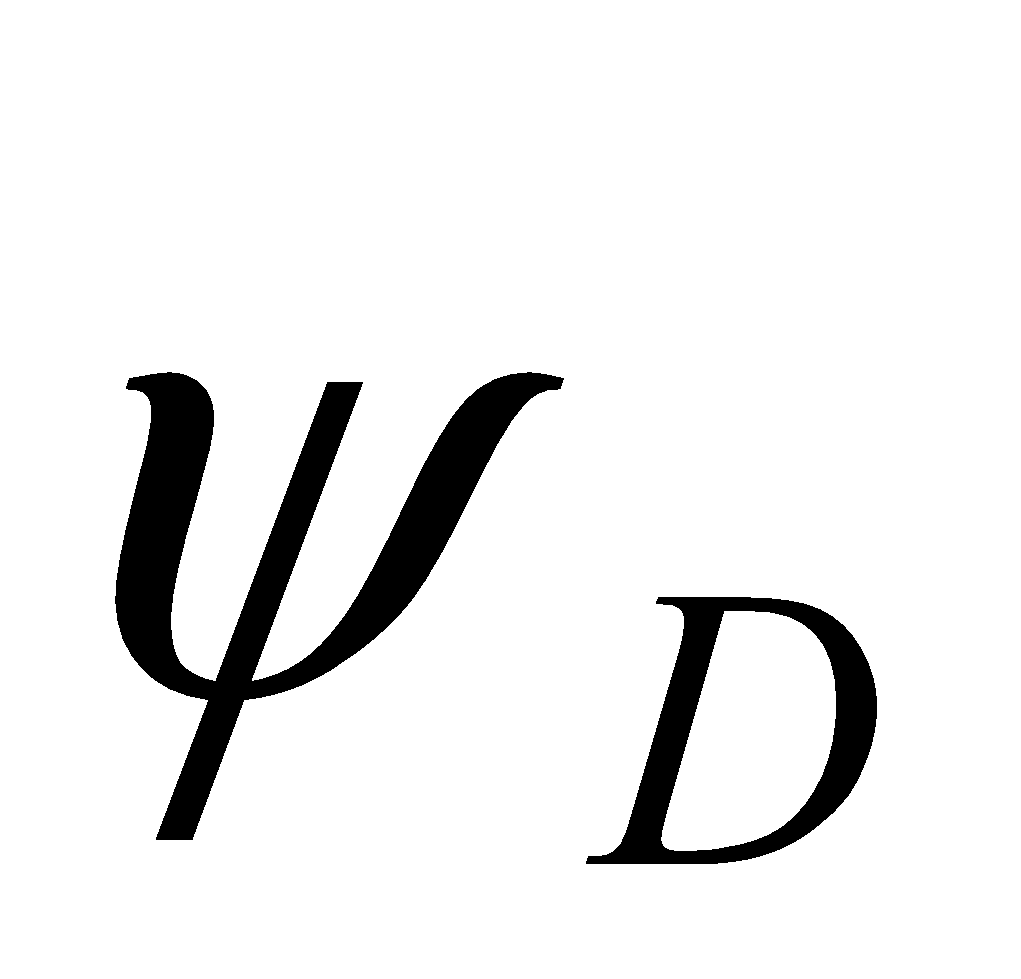
=
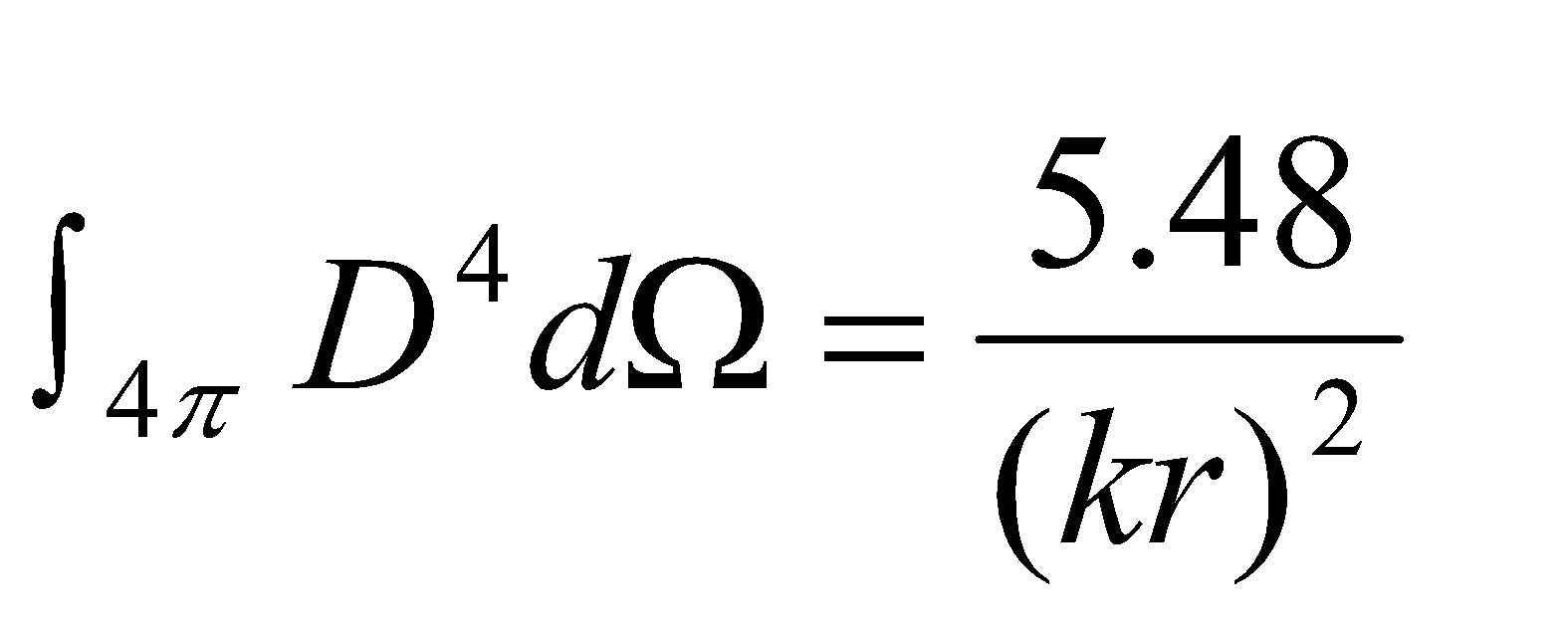
 (3)

where *D* is the amplitude beam pattern,
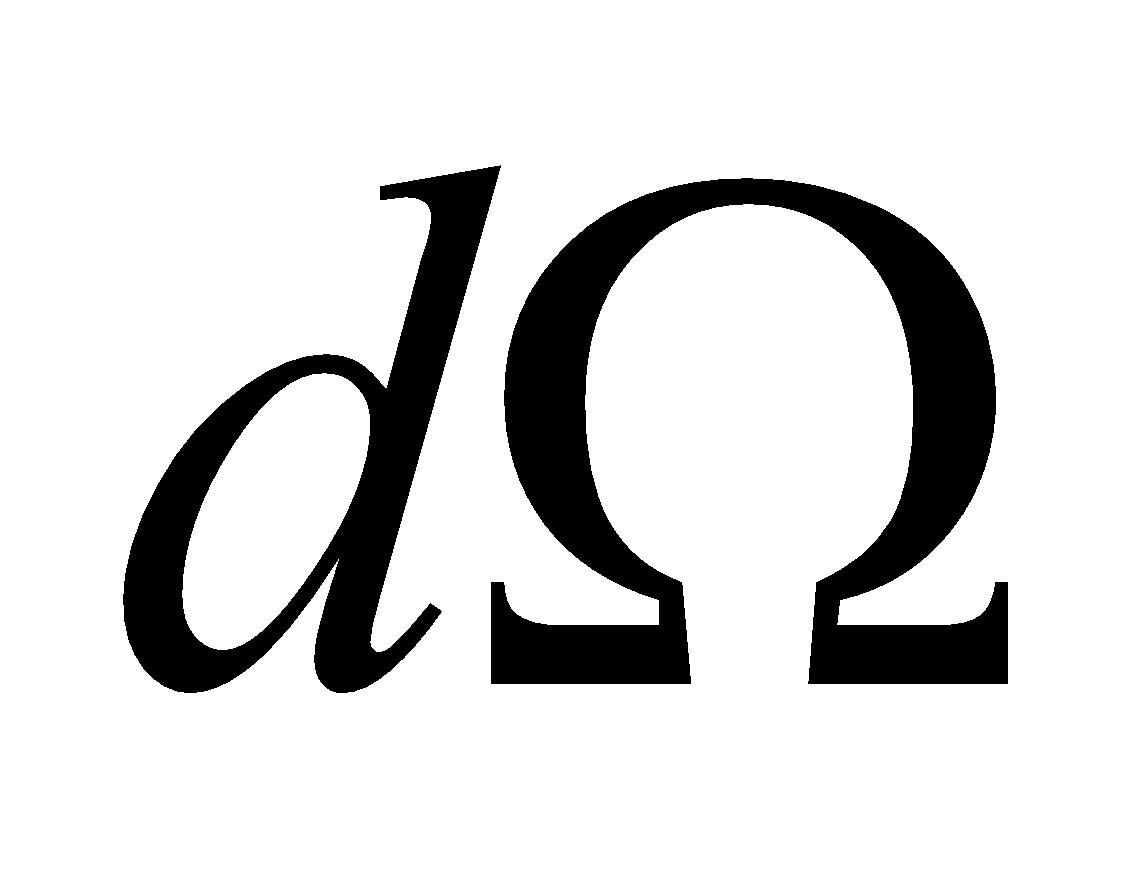
 is the solid angle increment, *k* is the acoustic wave number, and *r* is the transducer radius.

*TL* is the transmission loss, which can be calculated as follows:

*TL*=2
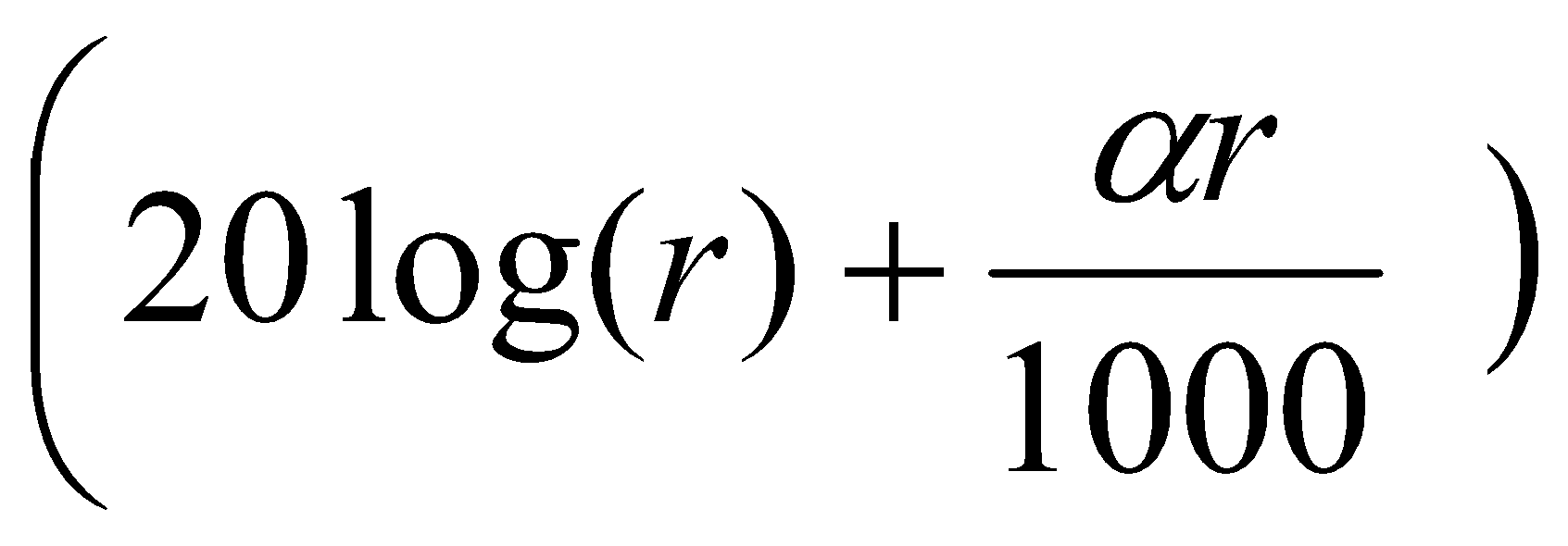
 (4)

where *r* (in meters) is the distance between the acoustic projector and the scattering bubbles and
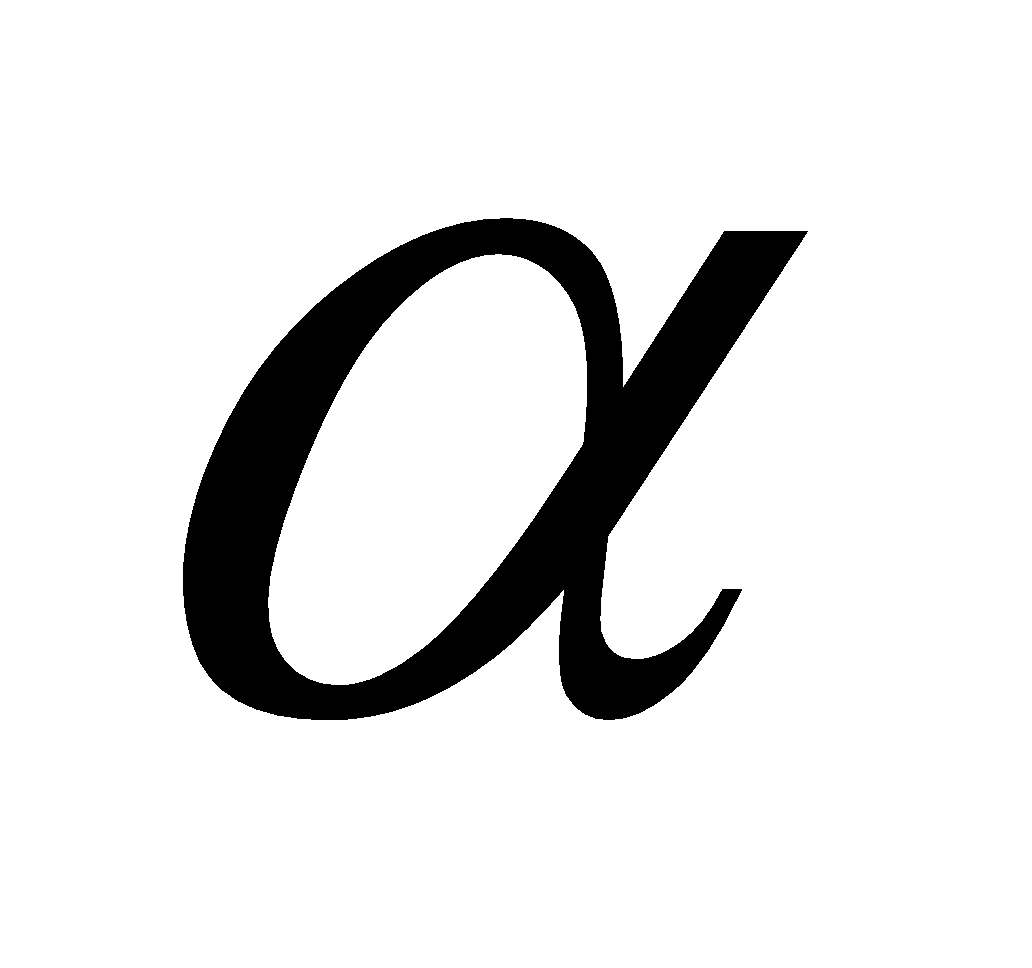
 is the attenuation coefficient of the sea water in dB per 1 km. *RL* is computed from the relationship:

*RL(t)*= 10 log
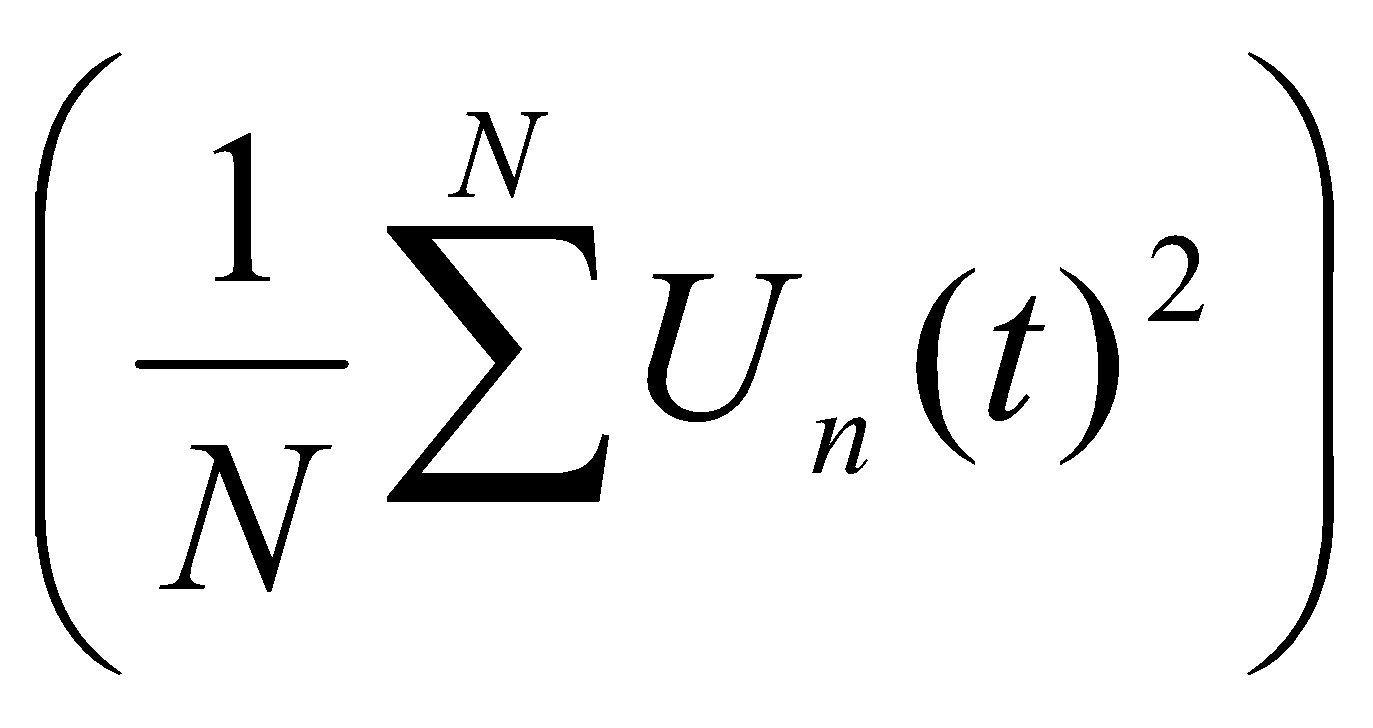
 (5)

where *U_n_*(t) is the received voltage for the individual ping *n,* and *N* is the number of pings. The average volume backscattering strength was estimated from 3-min-long time series (~1800 individual pings).

The acoustic backscattering cross section per unit volume of bubbles was estimated following [S1]:


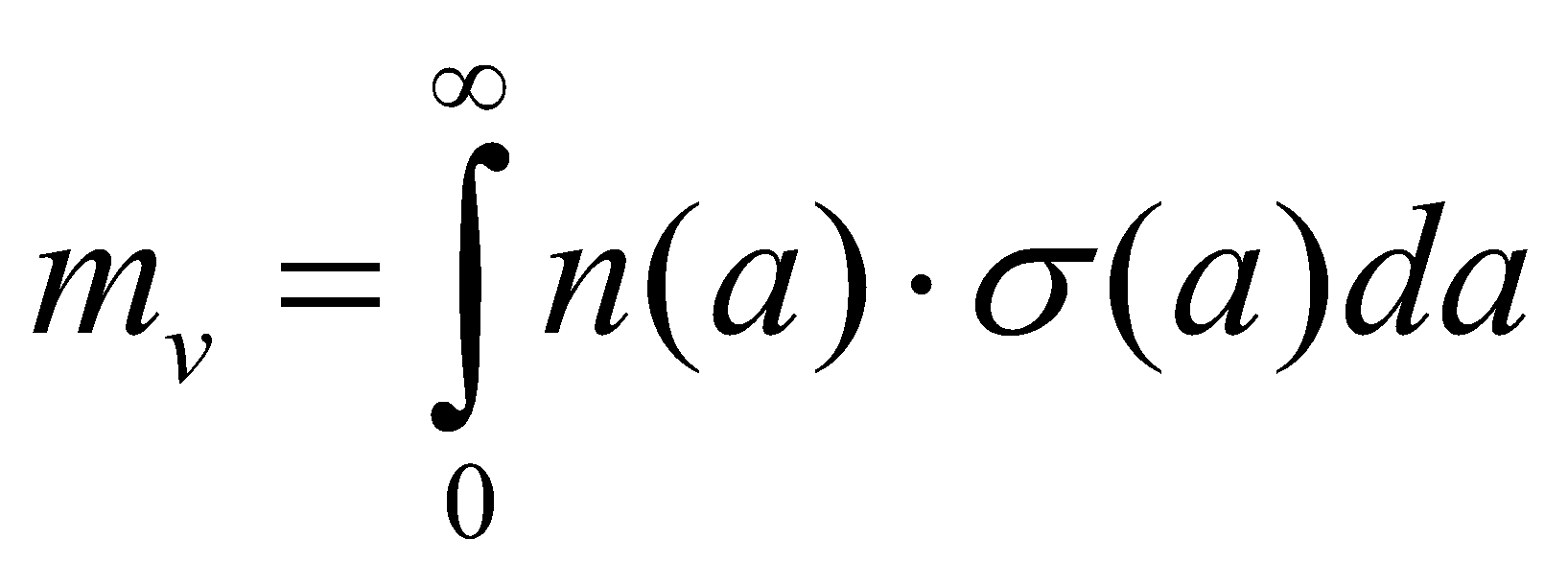
 (6)

where *n* is number of bubbles in the radius regime *a+Δa* and *Δa* = 1 µm. The scattering cross section, *σ,* of a bubble of radius *a* is:


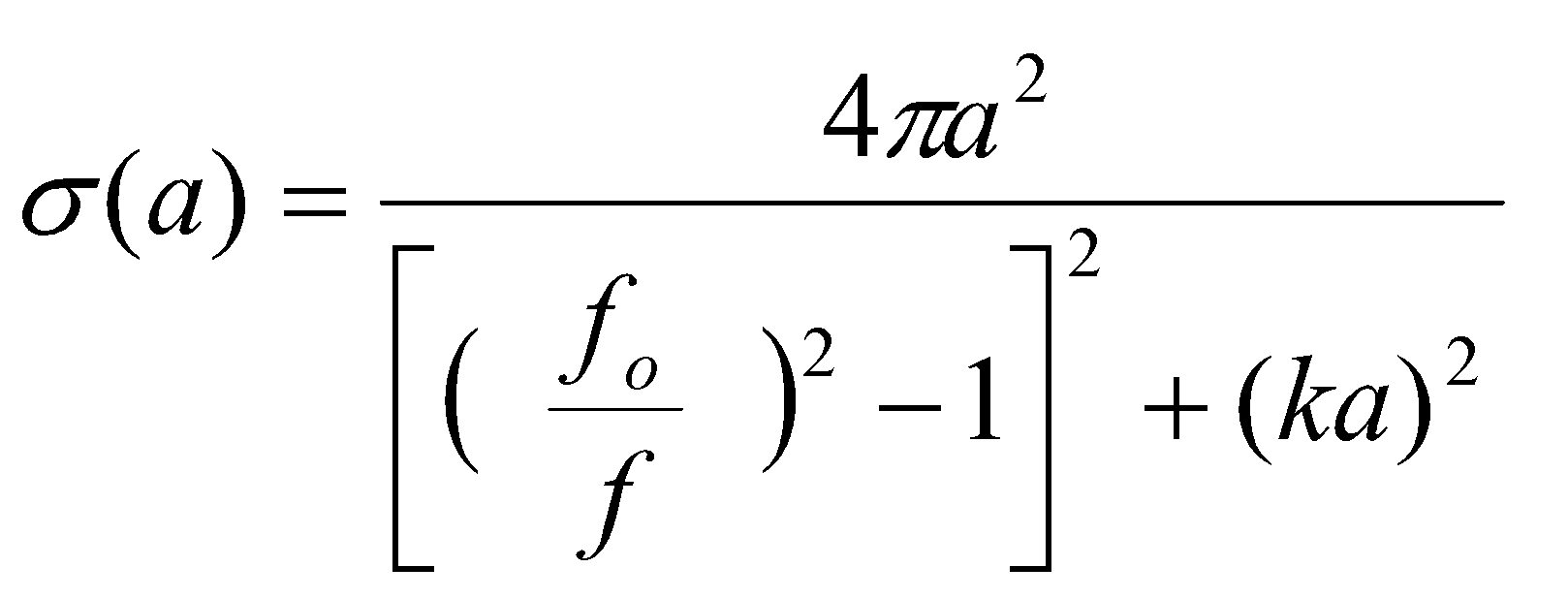
 (7)

where *k* is the acoustic wave number and *f_0_* is the approximate bubble resonance frequency derived from:


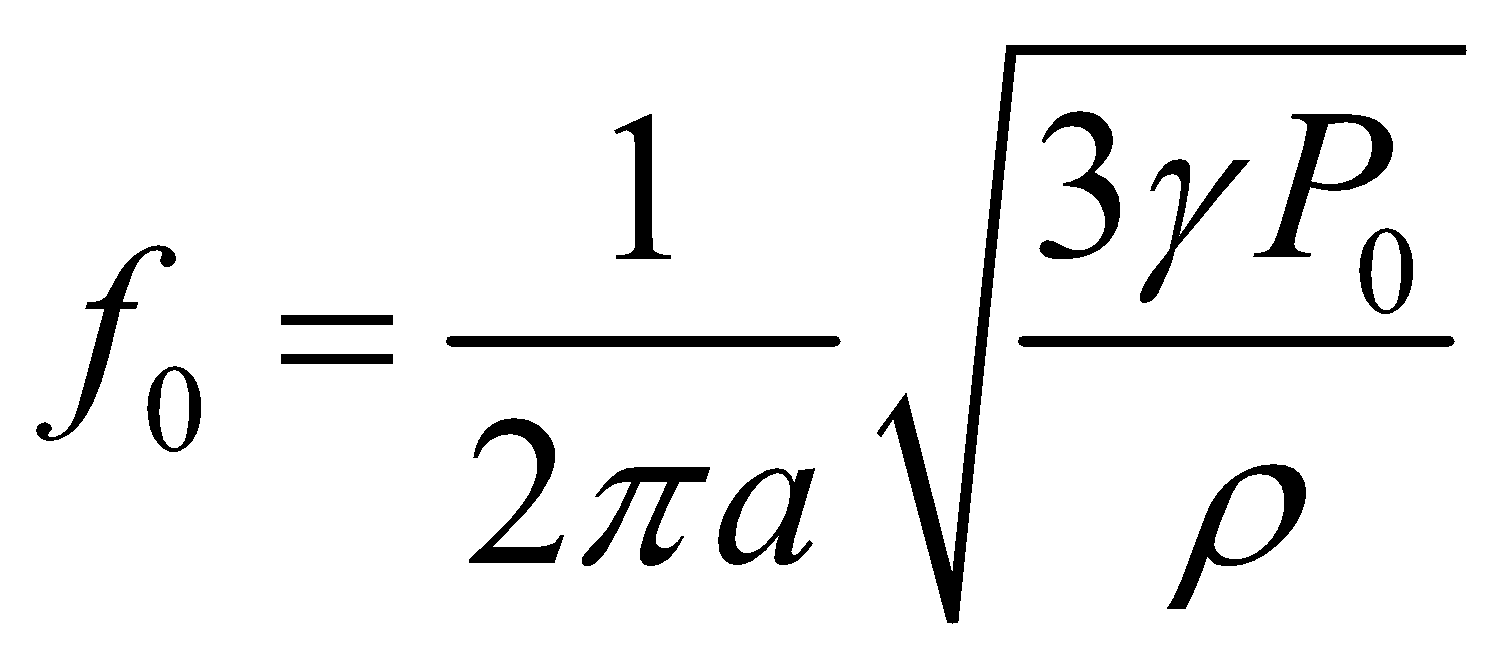
 (8)

where *γ* is the ratio of bubble gas specific heats, *P_0_* is the ambient pressure, and *ρ* is the ambient density of seawater.

Bubbles were observed during calm weather conditions using an ROV equipped with a high-speed high-resolution video camera. Using the ROV we located a seep field area from which bubbles were released distinctly one by one, which allowed us to take a series of pictures of each bubble using the high-speed high-resolution video camera. To resist currents and stabilize video recording and camera operation, the ROV was attached to a Rosette frame and the Rosette equipped with Niskin bottles was deployed to the sea floor and left in place for about 1 hour, which allowed us to determine bubble sizes relative to the sizes of particular parts of the Niskin bottles, which were marked with measuring tape. At each location bubbles were observed and recorded from 20 min to ~1 hour. Because bubble release frequency varied from 1.5 bubbles per second to 5.7 bubbles per second, 5400 to 36000 bubbles were available for analysis from each location.

Calculation of seep field density

To calculate how many flares existed in the P1 study area a correlation must be established between backscatter value and the numbers of acoustically-detected flares per m^2^ [S2, S3]. Because we detected seeps using the single-beam echo-sounder, to establish a correlation we considered the area that was actually insonified by the system. For example, the insonified area at 38 m water depth comprises 100 m^2^, while at 72.3 m water depth it is 380 m^2^. The size of the examined area was estimated as the total length of the vessel’s path over the sections of the examined area multiplied by the width of the zone sounded by the echo sounder (the diameter of the circle bounded by the beam width of the echo sounder transducer). It was assumed that all seep fields are round with a diameter that could be defined from the cross section of the flare observed on the echogram (*L*) using the equation [S4]:

D=L*
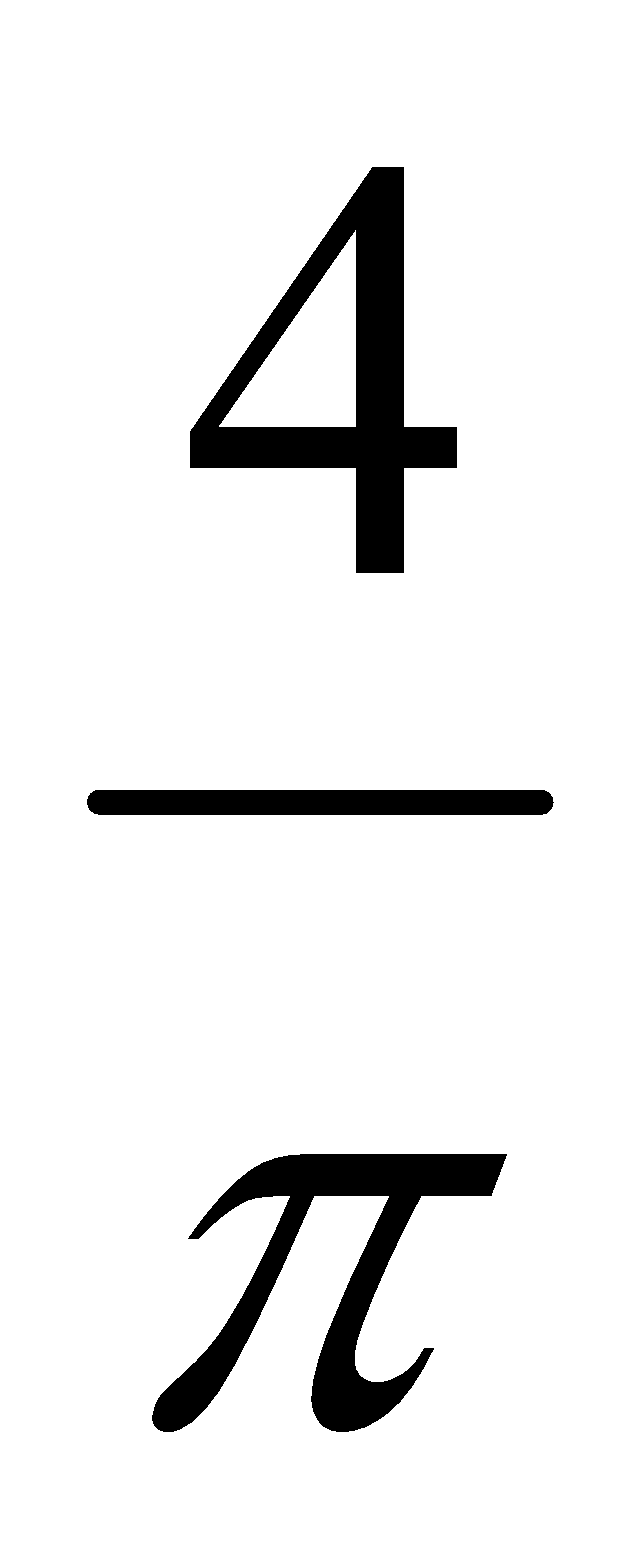
 (9)

Therefore, the area of the flare is:

S=
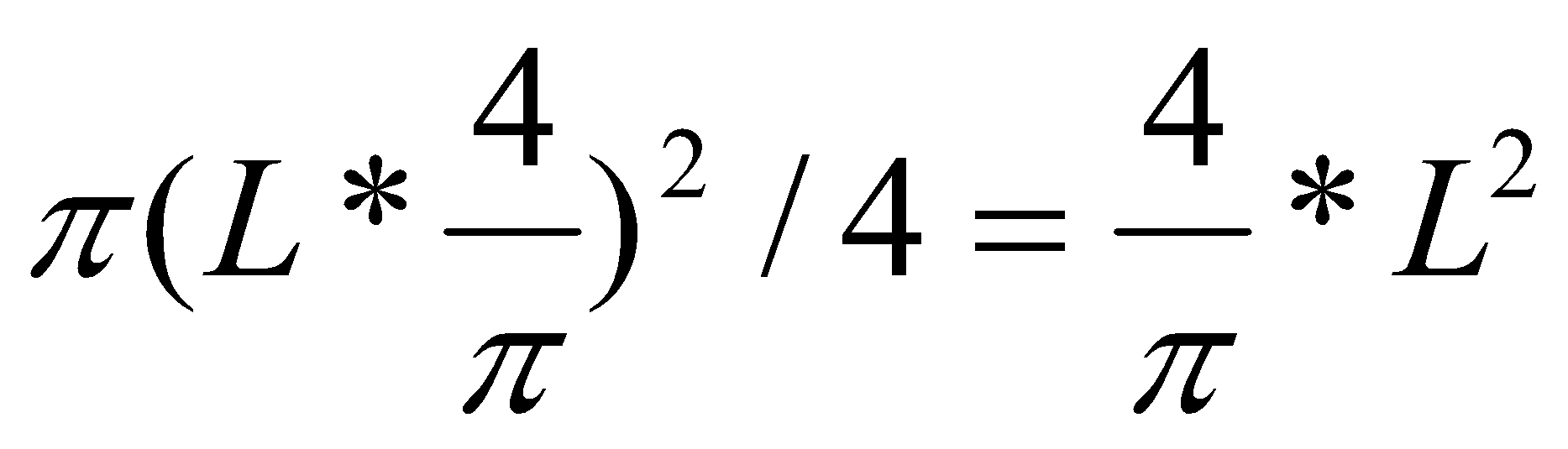
 (10)

The number of seep fields in a certain backscatter range (*i*) was divided by the field area to get the number of flares per m^2^:

*N_i_=n_i_**
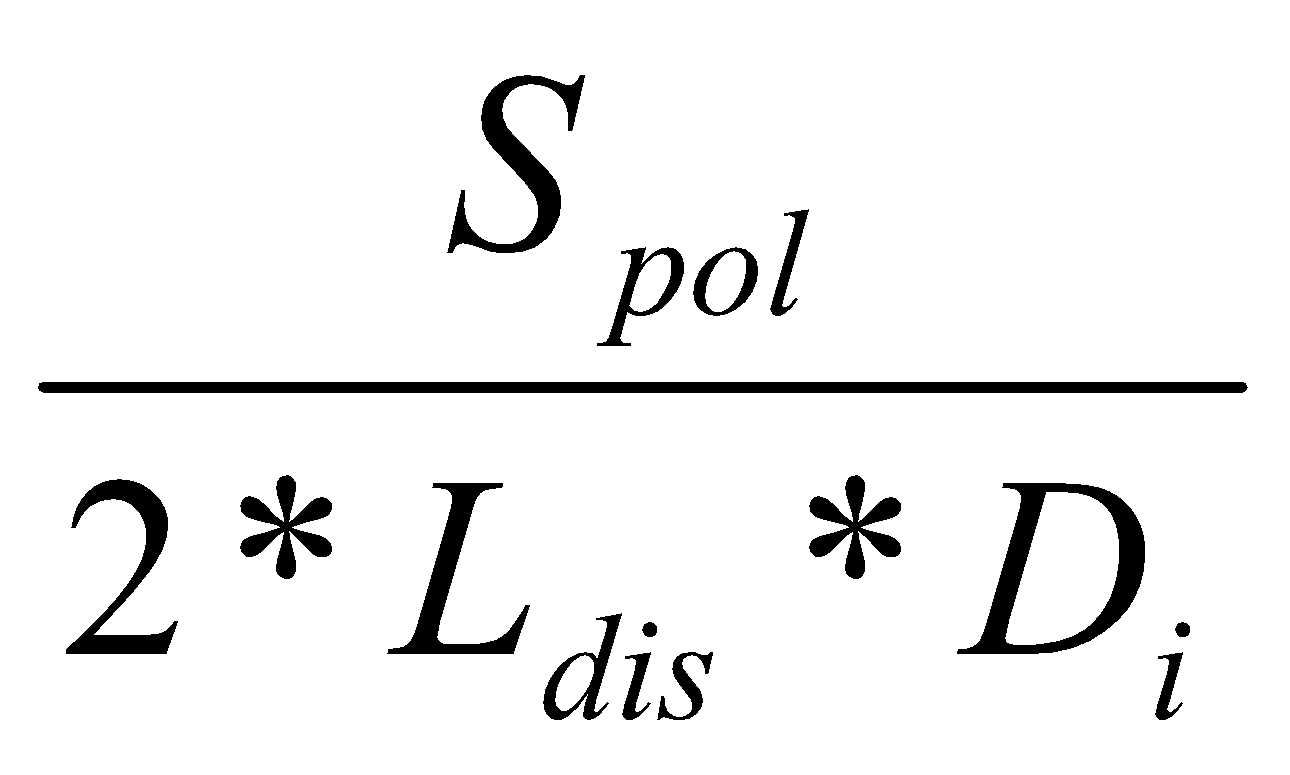
 (11)

Where *S_pol_* is P1 area*, n_i_* is the number of observed seep fields of a certain size, *L_dis_* is the vessel’s path length over the P1 section examined, and *D_i_* is the seep field diameter. Insonified areas in the F5 and F93 seep fields (Fig. S4) which were investigated in detail equaled 2.3 km^2^ (40% of 5.8 km^2^ total area) and 1.3 km^2^ (13% of 9.86 km^2^ total area), respectively, which allowed us to analyze 103 km (26 echograms, 60 minute each) for F5 and 67 km (23 echograms, 40 minutes each) for F93 (Fig. S4). The total number of P1 seep fields was determined by extrapolating the obtained seep field density to the total P1 area (Table S1 and S2).

Statistical testing of the data

To analyze the data set we created probability-probability plots (PPPs), which plot the two cumulative distribution functions against each other, to assess agreement among the data set variables. The PPPs show that the variables did not closely agree, indicating that different populations are represented in the data sets. The three resolved populations of small, medium, and large flare seep fields were tested using an empirical distribution function test in the Statistics 7.0 software package. The best fitting function was lognormal distribution. For the uncensored (i.e. all detects) datasets, the Maximum Likelihood (ML) estimates [S5] of the mean (*m**) and variance (*s*^2^*) were calculated using equations (1) and (2), respectively:


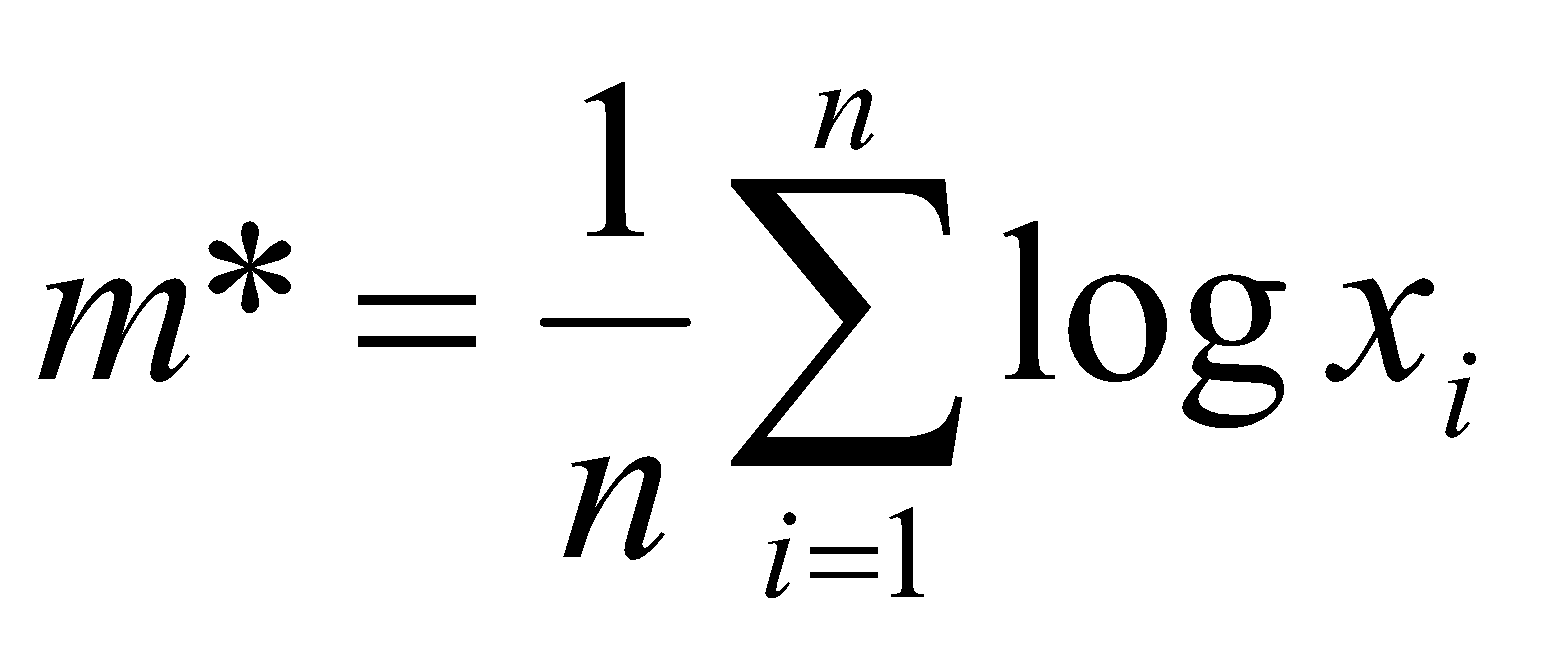
 (12)

where *n* is the number of observations and *x_i_* is the flare area or methane (CH_4_) flux (Table S1), and


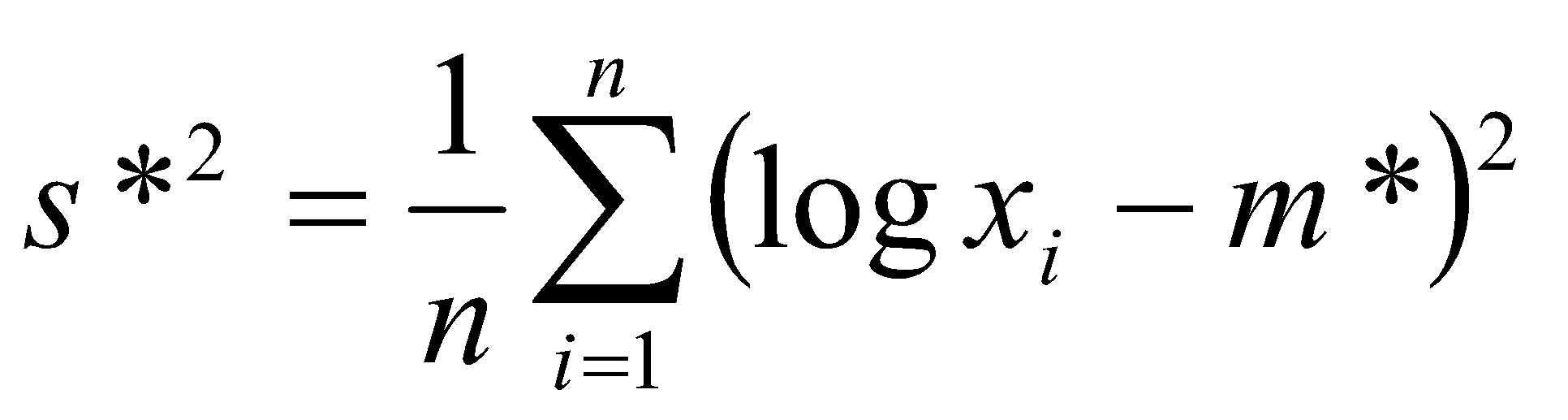
 (13)

The parameters *m** and *s*^2^* were then used to estimate the arithmetic mean and median of the lognormal distribution. For the log-normally-distributed population, the arithmetic mean (a) was estimated using:


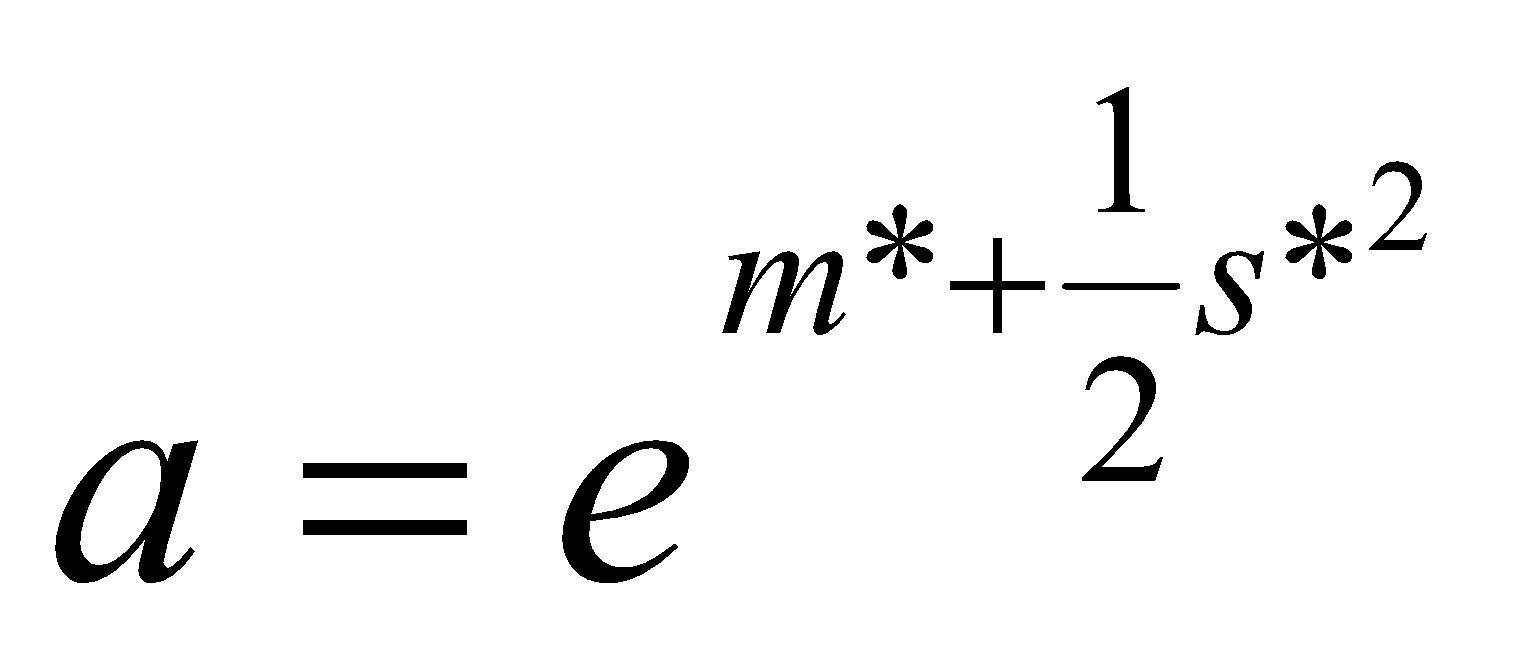
 (14)

From the definition of the arithmetic mean of a lognormal distribution, it is seen that this value is always greater than zero. This property guarantees that confidence interval estimates always attain positive values. This is a major advantage compared to normal mean confidence intervals, for which, in contrast, the lower bound of the confidence interval can be assigned a nonsensical negative value. Because of the positive skewness of the lognormal distribution, the lognormal a confidence interval is also positively skewed. The medians of the log-normally-distributed datasets were estimated using equation:


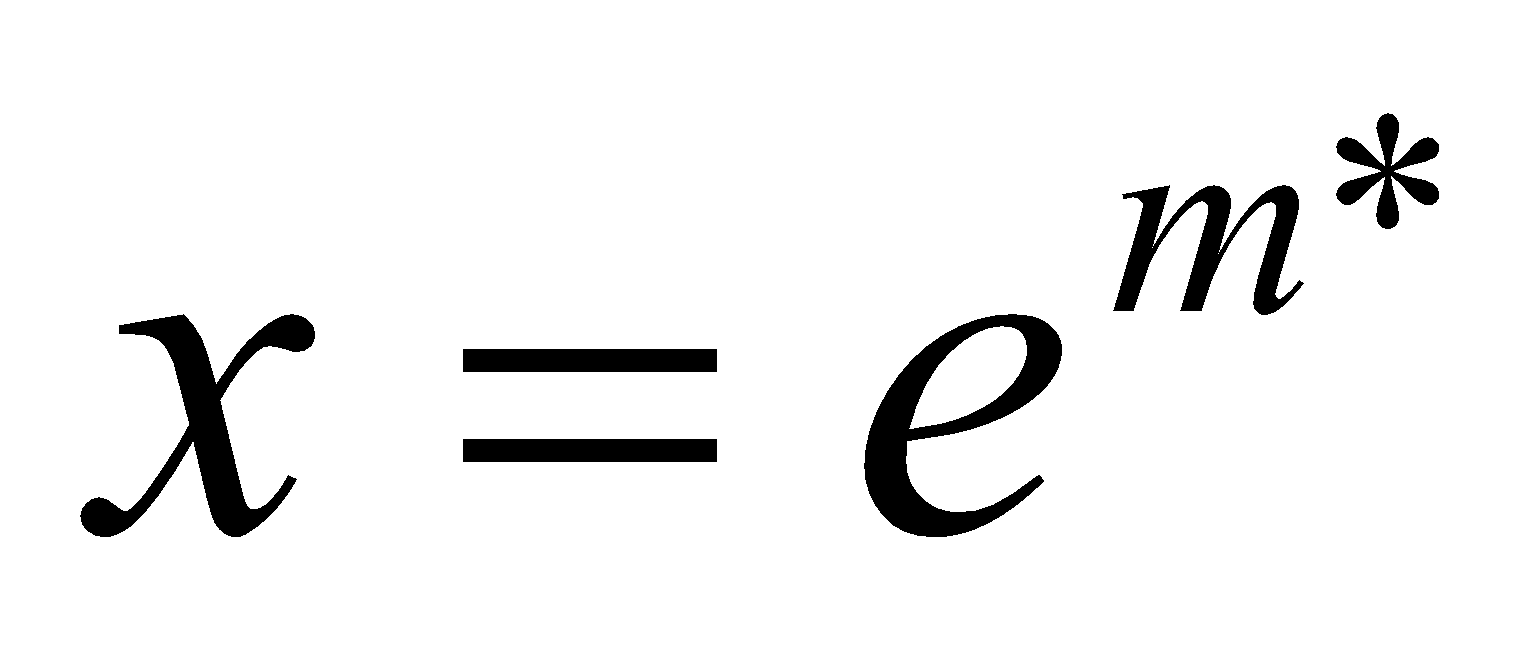
 (15)

Bubble fraction reaching the atmosphere as determined by in-situ observations

To assess what fraction of bubble-borne CH_4_ reaches the sea surface, we performed experimental work from the fast ice in April 2013 in the southern part of P2. Because bubble collection at sea is an extremely challenging task, especially in the open water, we chose to work from the fast ice in winter, when seawater is calm, visibility is better than in summer, and the sea ice prevents bubble escape to the atmosphere, which makes bubble collection feasible. We drilled a hole in the sea ice and created an engineered seep at ~6 m water depth; a gas tank was installed on the fast ice. By tuning the valve (changing the pressure) installed on the gas tank head, bubble flow controlled by flowmeter was changed from 0.2 to 2.0 liter per minute, creating a flow of ~5 mm diameter bubbles. Then we captured bubbles escaping from the water surface using a chamber installed over the hole in the sea ice. After a 1-hour exposure, we examined the composition of gas collected in the chamber and thus measured actual CH_4_ flux from the water surface. Our data show that at shallow water depth, ~67-72% of CH_4_ remains in the bubbles when the bubbles reach the sea surface (Table S3).

To visually examine and measure bubble sizes in one of the hot spots detected earlier, which exhibited high concentrations of CH_4_ in the surface water in both summer and winter, we drilled a hole in the fast ice and observed and recorded bubbles using the ROV deployed from another hole (3 m from the first hole) and then allowed thin ice (4-6 cm) to form during the night (air temperature at night was from -40̊C to -20̊C) so bubbles could not escape to the atmosphere. That allowed bubble sizes to be observed by naked eye from above the ice and quantified using a measuring tape. Observed individual bubble radii ranged from 2 to 12.5 mm. Thus, winter data revealed greater variability in bubble radii than that observed during the summer investigation at sea. This could be due to the specific features of bubbles rising in shallow water beneath the sea ice - bubbles merge creating bubbles of greater size instead of dissolving, as can be clearly seen in the video recording.

Methane oxidation rates

CH_4_ oxidation rates were measured in the water column, using a C_3_H_4_ radiotracer [S6, S7]. Gas-tight 10 ml syringes (*n* = 3 live samples plus 1 filter-sterilized control per depth) were filled to overflowing at each depth with water from a Niskin bottle. The syringes were sealed by inserting a plunger into the barrel and closing the hub end with modified luer lock tips fitted with gas-tight butyl rubber septa. A C_3_H_4_ stock solution was prepared by equilibrating 38 ml of filtered, degassed Mono Lake water with 1 ml of C_3_H_4_ in a gas-tight serum bottle. A 100–200 ml aliquot of C_3_H_4_ tracer solution was injected into each syringe through the butyl septum in the luer tip, yielding a tracer activity of ~30 000 dpm ml^-1^. Sample syringes were incubated at in situ temperature for 48 h. Rates were linear for more than 48 h (checked during the incubation period; data not shown). Incubations were terminated by dispensing the contents of each syringe into a glass scintillation vial containing an NaOH pellet, which halted biological activity. Labelled C_3_H_4_ was expelled by purging the sample with water-saturated CH_4_ for 5 min, vortexing, and venting the vials on a shaker table (50 r.p.m. for 30 min). Scintillation cocktail (Scintiverse BD, Fisher Scientific) was added and samples were counted for ^3^H_2_O activity using a Beckman 6500 liquid scintillation counter. The CH_4_ oxidation rate constant was calculated following [S7] and multiplied by in-situ CH_4_ concentration to determine CH_4_ oxidation rate.

Modeling lateral transport of dissolved CH_4_ on the East Siberian Arctic Shelf (ESAS)

The climatological circulation in the Arctic Ocean during 1997-2006 was reconstructed by assimilating oceanic and surface heat data into the Semi-Implicit Ocean Model [S8, S9] with 26×26 km resolution using the four dimensional variational (4Dvar) approach [S10]. Several kinds of data were used: 1) monthly temperature/salinity climatology was estimated by averaging available observations from the Arctic and Antarctic Research Institute database over the model grid points; 2) monthly averaged ocean-atmosphere and ocean-ice surface momentum and heat and salt fluxes from the Pan-Arctic Ice-Ocean Modeling and Assimilation System (PIOMAS) [S11] were used as atmospheric data; 3) the ice velocity and concentration from <http://nsidc.org/data/seaice> were used to constrain surface ocean momentum fluxes; and 4) data on monthly averaged Bering Strait transport were also assimilated. A similar approach was applied to reconstruct Chukchi Sea circulation during 1990-1991, which is described in detail in [S8]. The reconstructed annual velocities at 12.5 m water depth were used to calculate the trajectories of several groups of Lagrangian particles (molecules of dissolved CH_4_) using the standard Runge-Kutta fourth order accuracy algorithm [S9]. Particles were launched in three areas of the ESAS (the Laptev Sea, the East Siberian Sea, and the Chukchi Sea). Transport of the particles was integrated for a period of two and three years, which is in accordance with turnover time of the dissolved CH_4_ pool in the ESAS.

Permafrost modeling

We employed the recently developed general thermodynamic model [S12] of the permafrost evolution in ESAS that accounts for the duration of the transgression/regression cycle, air temperature, temperature of the ocean bottom water, the geothermal heat flux, thermal properties of the ground material, and salinity of the pore water in sediments. A modification of the model that has been described in detail [S12] was performed as a case study. The model parameters such as the thermal conductivity, heat capacity, porosity, and salinity of the ground material were set using observational data collected during the drilling campaigns performed in the near-shore area of the ESAS in 2009-2013. Recently obtained data on temperature dynamics of bottom water during the last 14 years (1999-2012) were employed to update the historical data set and to determine the thermodynamic state of sediments over decadal and multi-decadal time scales [described in details in S13]. Thermal conductivity and the heat capacity of the ground material were parameterized as functions of ice, liquid water, and salt concentration (values of key parameters established from field data for the studied area). The thermodynamic model was forced by seawater temperature dynamics computed by global circulation models (GCMs). Since GCMs provide coarse-resolution temperature dynamics, we incorporated local seawater warming effects. We used a 1-D realization of the thermodynamic model (the size of a computational domain is 100 meters). The initial temperature distribution was set to measured values collected during drilling. To compute temperature dynamics at sites within tectonics fault zones we utilized a 2-D realization of the thermodynamic model, which allows the formation and evolution of an open talik to be simulated. The computational domain and heat fluxes at its boundaries were determined as described in [S12].

Supplementary Tables

**Table S1.** Summary of parameters used for CH_4_ flux calculation

|  | **C^a^** | **IQR^b^** | **M^c^** | **CI^d^** | **M_l_^e^** | **M_u_^f^** |
| --- | --- | --- | --- | --- | --- | --- |
| Calculated area of an SF seep field, m^2^ | 52.83 | 90.99 | 85.25 | 39.73 | 65.38 | 105.11 |
| Calculated area of an MF seep field, m^2^ | 5567.2 | 7472.41 | 22459.9 | 44631.7 | 144.02 | 44775.7 |
| Calculated area of an LF seep field, m^2^ | 6358.5 | 549799.0 | 41998.4 | 87520.9 | 0 | 87520.9 |
| Calculated flux from an SF seep field, mol/s | 0.001 | 0.002 | 0.0018 | 0.0087 | 0.0014 | 0.0023 |
| Calculated flux from an MF seep field, mol/s | 0.353 | 0.82 | 1.079 | 1.867 | 0.145 | 2.013 |
| Calculated flux from an LF seep field, mol/s | 0.364 | 0.874 | 3.632 | 8.955 | 0 | 8.955 |

^a^ median of a lognormal distribution; ^b^ inter-quartile range (i.e. third quartile minus first quartile); ^c^ arithmetic mean of a lognormal distribution; ^d^ confidence interval of the mean (P=0.95, alpha=0.05); ^e^ the 95% lower confidence limit of the mean; ^f^ the 95% upper confidence limit of the mean. SF: small flare, MF: medium flare, LF: large flare.

**Table S2.** Summary of flare seep field class parameters observed in the outer shelf in the ESAS

| Seep field class | Mean area, m^2^ | Mean diameter, m | Total number in P1 | Total P1 area, km^2^ | CH_4_ flux , mol/m^2^/d | Total CH_4_ flux from flare class, mol/d  *×* (10^6^)* |
| --- | --- | --- | --- | --- | --- | --- |
| SF seep field | 85.25 | 10.4 | 58240 | 4.9 | 1.9 | 9.3 |
| MF seep field | 22459.88 | 84 | 2560 | 57.5 | 5.5 | 316.2 |
| LF seep field | 41998.41 | 243 | 1920 | 80.8 | 11.0 | 888.8 |

*Total flux from the sea floor to the water column from three flare seep classes is 1214.3×10^6^ mol CH_4_ per day, which is 7×10^12^ g (7 Tg) CH_4_ per year.

**Table S3.** Gas composition of bubbles reaching the sea surface in P2.

| Statistics | O_2_, % | N_2_, % | CH_4_, % | CO_2_, % |
| --- | --- | --- | --- | --- |
| Mean (n=20) | 6.24 | 23.36 | 69.56 | 0.76 |
| Standard deviation | 1.24 | 4.28 | 5.67 | 0.5 |
| Confidence interval (P≤0.05) | 0.54 | 1.84 | 2.48 | 0.21 |
| 95% lower confidence limit of the mean | 5.7 | 21.52 | 67.08 | 0.55 |
| 95% upper confidence limit of the mean | 6.78 | 25.2 | 72.04 | 0.97 |

Supplementary Figures


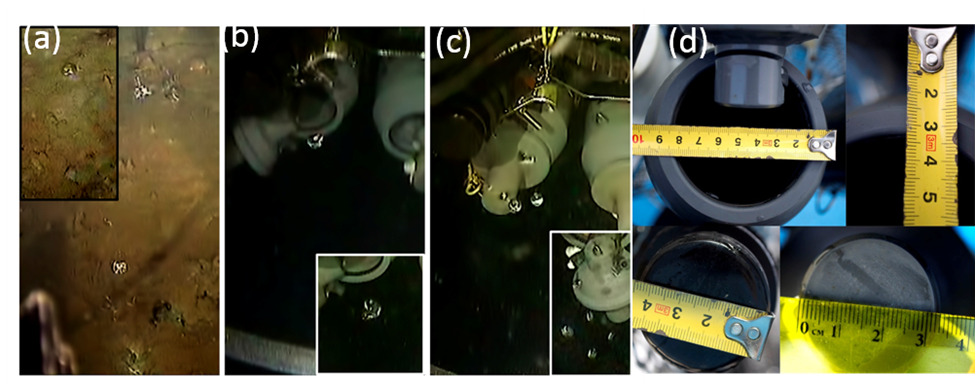


**Figure S1**. Results of in-situ bubble video-recording. (a) As seen in the panel, bubbles escape from the black holes (vents) in the seafloor; (b) and (c) show bubbles captured by the Niskin bottles installed on the Rosette; (d) presents dimensions of the Niskin bottle parts used as an in-situ scale to estimate bubble sizes.


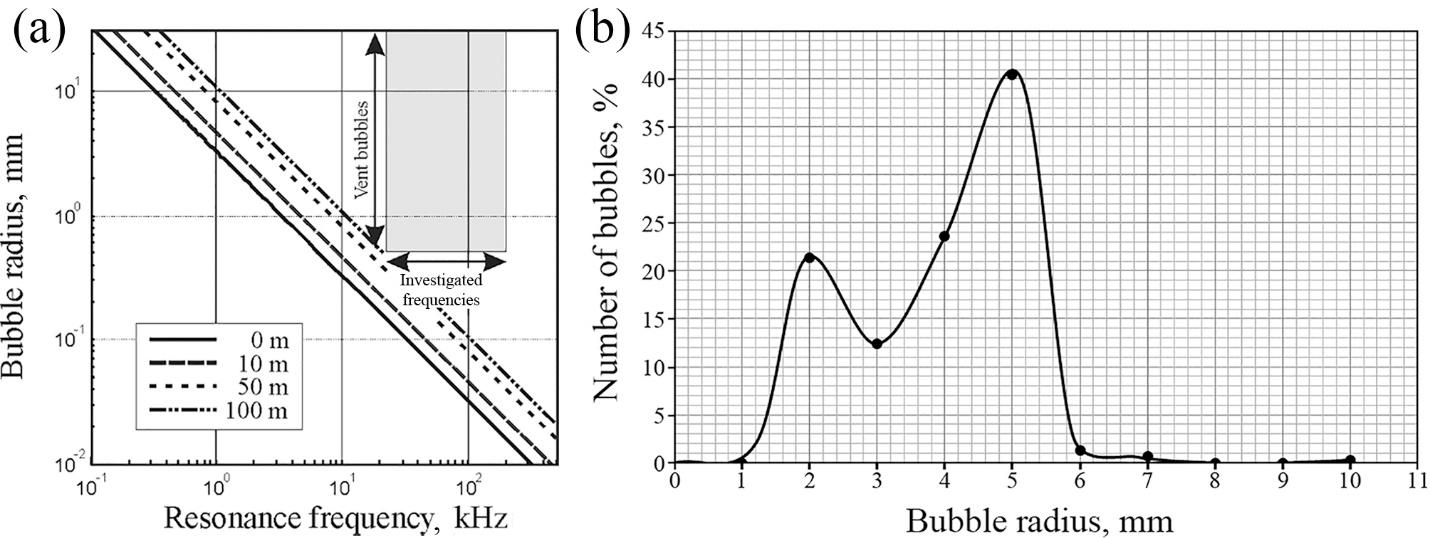


**Figure S2.** Results of bubble analysis. (a) Resonance frequencies as a function of bubble radius for four discrete depths [S5]. (b) Distribution of bubble radii in the observed population of bubbles. The grey area in panel (a) illustrates the frequencies associated with the bubble radii observed during the study (between 1 and 10 mm). To exclude a possible resonance effect, a frequency of 200 kHz was used for quantitative analysis.


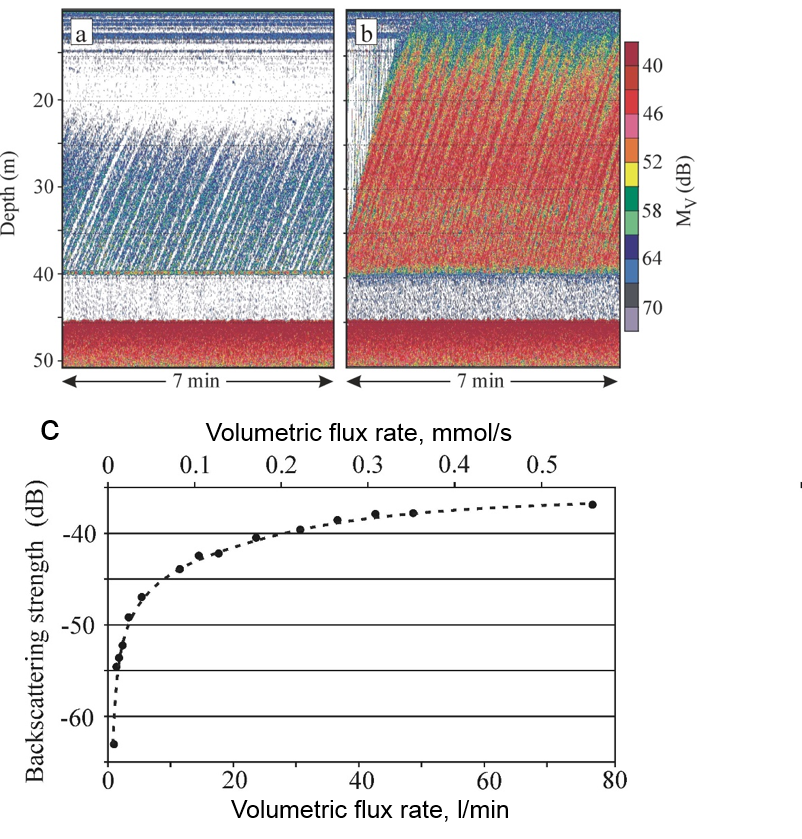


**Figure S3.** In-situ sonar calibration data. Examples of (a) an echogram recorded at a flux rate of 1.6 l/min; (b) an echogram recorded at a flux rate of 76.2 l/min. (c) The calibration curve was created as an empirical fit based on observed backscattering strength measurements at a frequency of 200 kHz. The data show a very good correlation between the volumetric flux rate and the strength of the backscattering signal (dB).


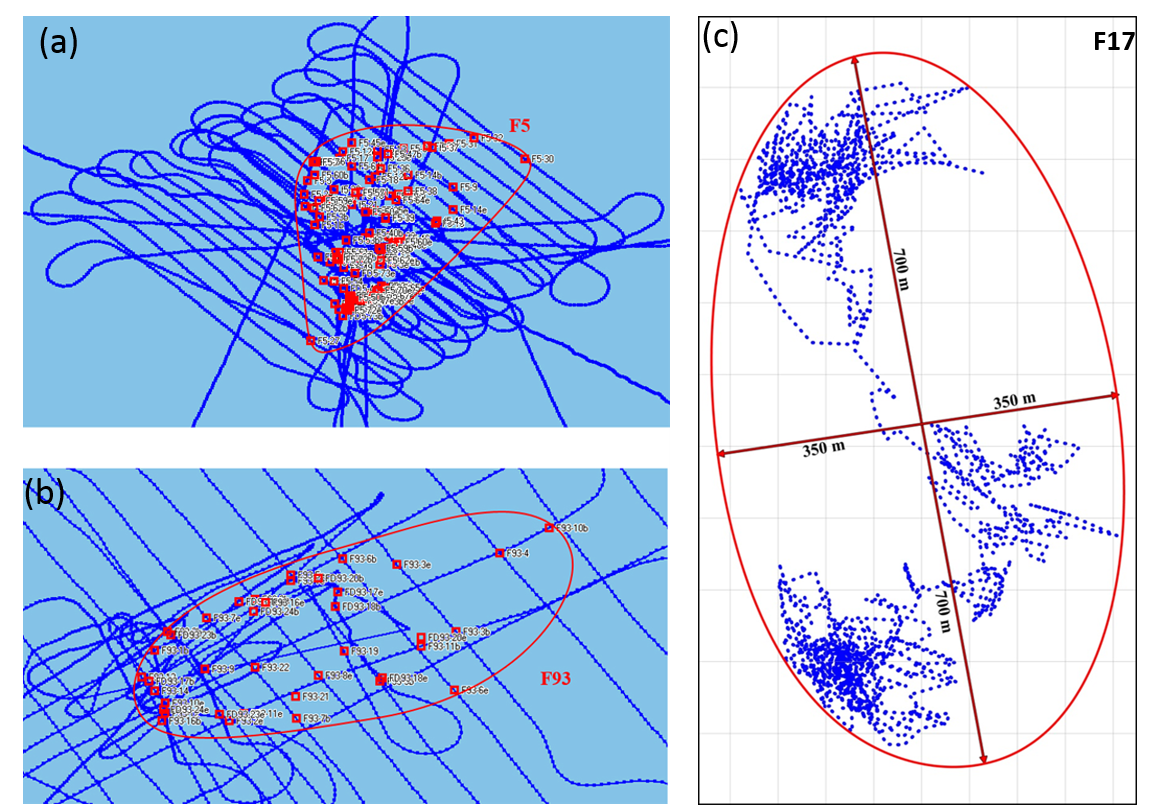


**Figure S4.** Schematic of ship tracks for hydro-acoustical surveys performed in P1 seep fields and occurrence of seeps within the seep fields. (a) Ship track in seep field F5, where 2599 distinct seeps were observed (total seep area 2.3 km^2^). (b) Ship track in seep field F93; 2272 distinct seeps were observed (total seep area 1.3 km^2^). (c) Seep density observed in the F17 seep field. These surveys aimed to establish a correlation between backscatter value and the numbers of acoustically detected seeps per m^2^. The size of the examined area was estimated by multiplying the total length of the vessel’s path over the sections of the examined area times the width of the zone sounded by the echo sounder (the diameter of the circle bounded by the beam width of the echo sounder transducer).


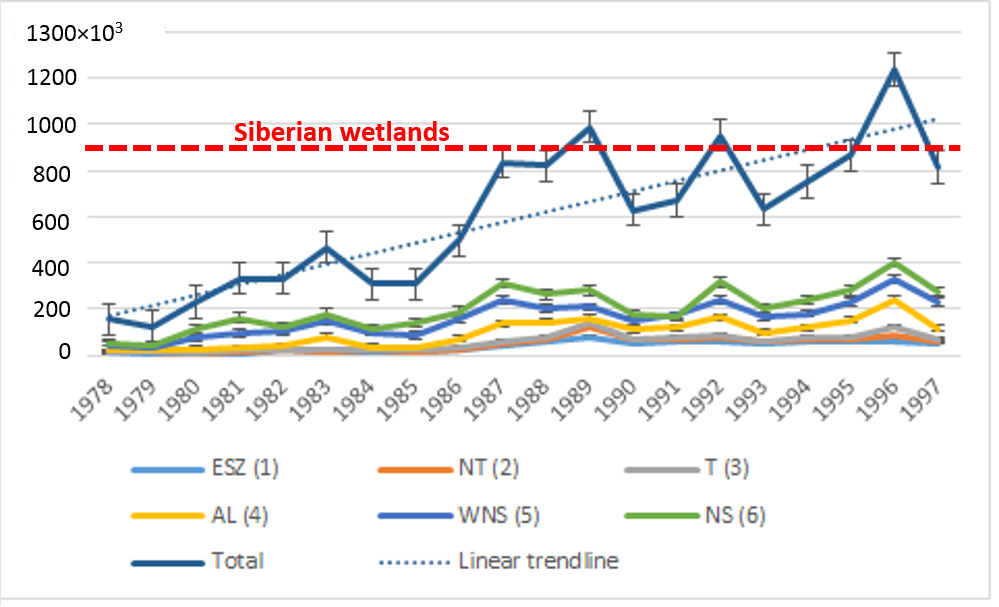


**Figure S5.** Increase in the areas of flaw polynyas composing the Great Siberian Polynya observed over two decades: ESZ – Eastern Severnaya Zemlya polynya, NT – Northeastern Taimyr polynya, T – Taimyr polynya, AL – Anabar Lena polynya, WNS – Western New Siberian polynya, NS – New Siberian polynya, after [S14]. An area of the Siberian wetlands is shown as red dotted line [S15].


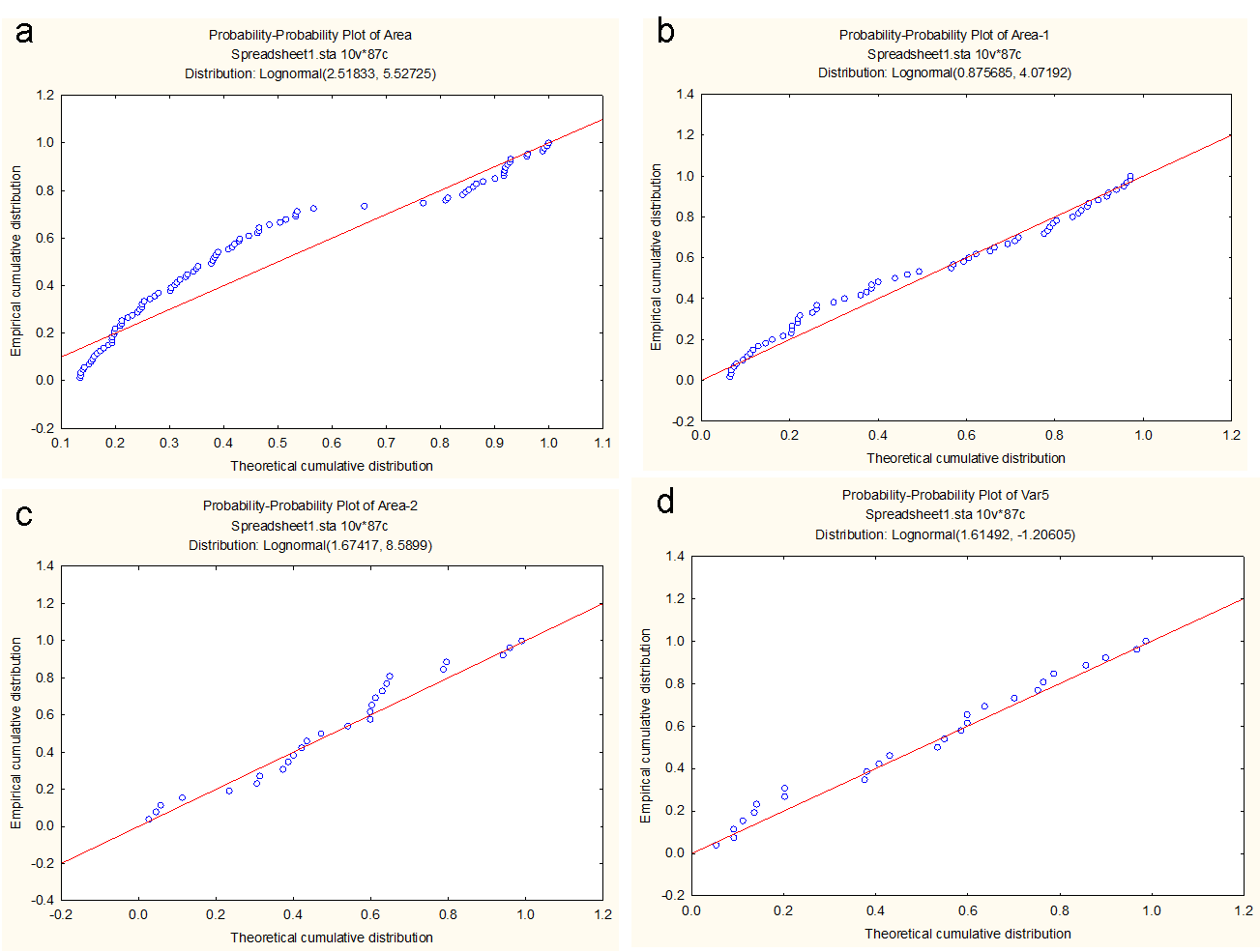


**Figure S6**. Probability-probability plots (PPPs) built for the entire population (a) and for three resolved sub-populations (b, c, and d). The PPP in panel (a) shows that variables did not closely agree, indicating that different sub-populations are presented in the data sets. The PPPs built for subpopulations of SF flare seep fields (b), MF seep fields (c), and LF seep fields (d) show better agreement between variables in each sub-population.

**Supplementary references.**

S1. Greinert, J., Nutzel, B. 2004 Hydro-acoustic experiments to establish a method for the determination of methane bubble fluxes at cold seeps. *Geo-Mar Lett* ***24***:75-85. (doi: . 10.1007/s00367-003-0165-7).

S2. McGinnis, D. F., Greinert, J., Artemov, Y., Beaubien, S.E., Wuest, A. 2006 Fate of rising bubbles in stratified waters: How much methane reaches the atmosphere? *J Geophys Res* ***111***:C09007. (doi:10/1029/2005JC003183).

S3. Greinert, J., McGinnis, D. F., Naudts, L., Linke, P., De Batist, M 2010 Atmospheric methane flux from bubbling seeps: Spatially extrapolated quantification from a Black Sea shelf area.  *J Geophys Res* ***115***:C01002. (doi:10.1029/2009JC005381).

S4. Salomatin A.S., Yusupov V.I. 2011 Acoustic Investigations of Gas Flares in the Sea of Okhotsk. Oceanology Vol. 51, 857–865. (doi: 10.1134/S0001437011050134).

S5. Jonsson, A., Gustafsson, O., Axelman, J., Suindberg, H. 2003 Global accounting of PCBs in the continental shelf sediments. *Environ Sci Technol* ***37***:245.

S6. Carini, S., Bano, N., LeCleir, G., Joye, S. B. 2005 Aerobic methane oxidation and methanotroph community composition during seasonal stratification in Mono Lake, California (USA). *Environ Microbiol* ***7***:1127-1138. (doi: 10.1111/j.1462-2920.2005.00786.x).

S7. Sandbeck, K. A, Reeburgh, W. S. 1989 Microbiological preparation of H-3-labelled methane. *Label Comp Radiopharm* ***2***7: 1285–1291.

S8. Panteleev, G., Nechaev, D., Ikeda, M. 2005 Reconstruction of summer Barents Sea circulation from climatological data. *Atmos Ocean*  ***44(2)***:111-132. (doi: 10.3137/ao.440201).

S9. Nechaev, D., Panteleev, G., Yaremchuk, M. 2005 Reconstruction of the circulation in the limited region with open boundaries: circulation in the Tsushima Strait. *Okeanologiya* ***45(6)***:805-828.

S10. Wunsch, C. 1996 *The Ocean Circulation Inverse Problem* (Cambridge Univ. Press, Cambridge) 442 pp.

S11. Roache, P. J. 1998 *Fundamentals of Computational Fluid Dynamics*. (Hermosa Publishers, Albuquerque, N M) 648 pp.

S12. Nicolsky, D. J., et al. 2012 Modeling sub-sea permafrost in the East Siberian Arctic Shelf: The Laptev Sea region. *J Geophys Res* ***117***:F03028. (doi:10.1029/2012JF002358).

S13. Shakhova, N., et al. 2014 Ebullition and storm-induced methane release from the East Siberian Arctic Shelf. *Nature Geosci* **7(1):**64-70. (doi:10.1038/NGEO2007).

S14. Zakharov, J. M. 2003 Changes of the Arctic sea ice extent in XX century. Meteorologiya I Gidrologiya 5, 75-86.

S15. Kremenetski, K. V. et al. 2003 Peatlands of the Western Siberian lowlands: current knowledge on zonation, carbon content and Late Quaternary history. *Quaternary Science Reviews* **22**, 703–723.
